# Oral and Injected Tamoxifen Alter Adult Hippocampal Neurogenesis in Female and Male Mice

**DOI:** 10.1523/ENEURO.0422-21.2022

**Published:** 2022-04-19

**Authors:** Bryon M. Smith, Angela I. Saulsbery, Patricia Sarchet, Nidhi Devasthali, Dalia Einstein, Elizabeth D. Kirby

**Affiliations:** 1 Department of Psychology, College of Arts and Sciences, The Ohio State University, Columbus, OH 43210; 2 Chronic Brain Injury Initiative, The Ohio State University, Columbus, OH 43210

**Keywords:** adult neurogenesis, hippocampal neurogenesis, hippocampus, inducible Cre recombinase, neurogenesis, tamoxifen

## Abstract

Inducible Cre recombinase facilitates temporal control of genetic recombination in numerous transgenic model systems, a feature which has made it a popular tool for adult neurogenesis studies. One of the most common forms of inducible Cre, CreER^T2^, requires activation by the selective estrogen receptor modulator tamoxifen (TAM) to initiate recombination of LoxP-flanked sequences. To date, most studies deliver TAM via intraperitoneal injection. But the introduction of TAM-infused commercial chows has recently expanded the possible modes of TAM delivery. Despite the widespread use of TAM-inducible genetic models in adult neurogenesis research, the comparative efficiency and off-target effects of TAM administration protocols is surprisingly infrequently studied. Here, we compare a standard, 5 d TAM injection regimen with voluntary consumption of TAM-infused chow. First, we used adult NestinCreER^T2^;Rosa-LoxP-STOP-LoxP-EYFP reporter mice to show that two weeks of TAM chow and 5 d of injections led to LoxP recombination in a similar phenotypic population of neural stem and progenitor cells (NSPCs) in the adult dentate gyrus. However, TAM chow resulted in substantially less overall recombination than injections. TAM administration also altered adult neurogenesis, but in different ways depending on administration route: TAM injection disrupted neural progenitor cell proliferation three weeks after TAM, whereas TAM chow increased neuronal differentiation of cells generated during the diet period. These findings provide guidance for selection of TAM administration route and appropriate controls in adult neurogenesis studies using TAM-inducible Cre mice. They also highlight the need for better understanding of off-target effects of TAM in other neurologic processes and organ systems.

## Visual Abstract

**Figure:**
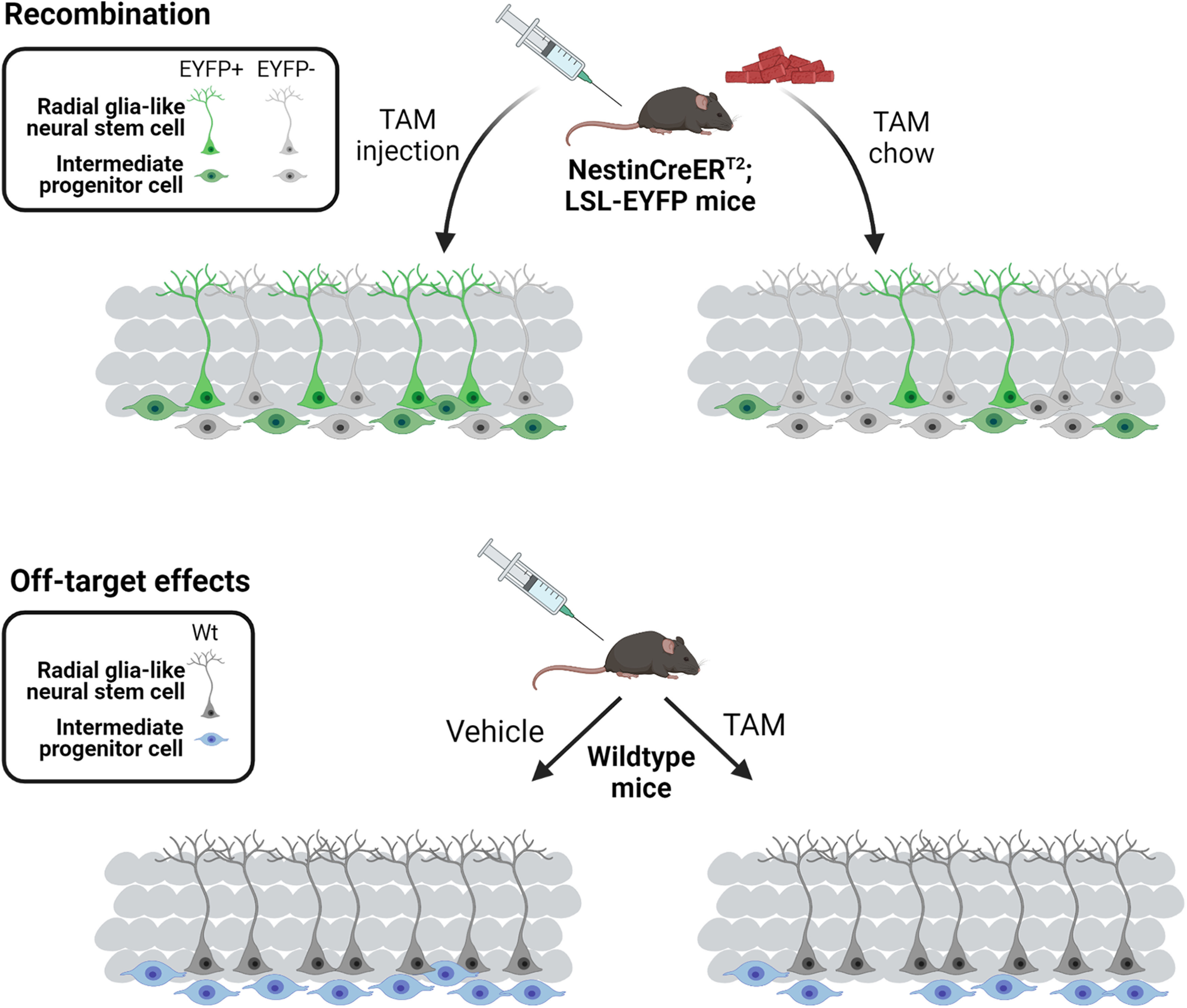

## Significance Statement

Numerous transgenic mouse models use the inducible Cre-Lox system in which a synthetic estrogen tamoxifen (TAM) is used to initiate recombination of genetic sequences flanked by LoxP sites. Despite widespread use, there is little data available about the effectiveness of different TAM delivery methods. Here, we show that giving adult mice in which inducible Cre is expressed in neural stem and progenitor cells (NSPCs) TAM via injection (the most commonly-used route) and chow both induce recombination in those target cells. However, chow delivery is far less efficient than injections and both delivery methods alter adult neurogenesis on their own when compared with vehicle-treated mice. These findings provide guidance for investigators choosing experimental design with inducible Cre systems.

## Introduction

Adult neurogenesis is a widely conserved process among mammalian species in which resident neural stem cells generate new neurons that integrate into mature circuitry throughout the lifespan. Evidence that neurogenesis in the adult rodent dentate gyrus of the hippocampus supports memory and affect regulation has spurred interest in both its natural functions across species, as well as its potential therapeutic applications in humans (for review, see McAvoy and Sahay, 2017; Miller and Sahay, 2019; Toda et al., 2019).

The study of time-specific and tissue-specific phenomena, such as adult hippocampal neurogenesis, has been accelerated by the introduction of several inducible methods of gene expression manipulation. Tamoxifen (TAM)-inducible Cre mouse lines (e.g., CreER^T2^) have proven to be particularly valuable tools for inducible manipulation of gene expression in the adult neurogenic lineage. In these models, Cre recombinase is fused to a mutated estrogen receptor (ER^T2^) which retains Cre in the cytoplasm until the receptor binds its ligand, the selective estrogen receptor modulator TAM (Feil et al., 1997; Danielian et al., 1998). TAM binding allows translocation of the mutated estrogen receptor and its fused Cre enzyme to the nucleus, where Cre can induce recombination of LoxP-flanked genetic sequences. Use of a tissue-specific promoter can further constrain CreER^T2^ expression to specific cell classes. For example, neural stem and progenitor cells (NSPCs) are commonly targeted using GLAST, Nestin, SOX2, or GFAP promoters (to name a few common versions; Semerci and Maletic-Savatic, 2016).

In adult neurogenesis studies using CreER^T2^-mediated LoxP recombination, two dominant approaches to selecting controls have emerged. One approach compares mice of different genotypes (Cre+/− or LoxP+/−), all of which are treated with TAM. A second approach is to compare mice of the same genotype treated with TAM versus those treated with vehicle. This latter comparison is predicated on TAM itself being inert, aside from its ability to induce CreER^T2^ translocation. Yet, gonadal steroid hormones, including estrogens, can modulate brain processes such as neurogenesis and cell survival (Jorgensen and Wang, 2020).

The effect of TAM on adult neurogenesis is generally understudied and the few existing studies have yielded mixed results. For example, Rotheneichner et al. (2017) found no inherent effects of TAM on cell proliferation and fate in the dentate gyrus of the adult mouse hippocampus, but a more recent study suggested long-lasting suppression of hippocampal cell proliferation in juvenile mice (Lee et al., 2020). Given the widespread use of TAM-inducible models in adult neurogenesis, it is imperative to understand how TAM itself (independent of transgene expression) affects adult neurogenic processes.

Beyond the selection of controls, TAM administration route is another important potential design variable in studies using CreER^T2^ mouse models. Intraperitoneal injections are by far the most common method of exposing mice to TAM in adult neurogenesis studies to date. However, administration of TAM through voluntary consumption of custom laboratory chow is a promising alternative that might reduce injection-related stress and researcher hands-on time. TAM-infused chows have been used successfully in cardiac research (Kiermayer et al., 2007), and may be applicable to other adult tissues as well (Yoshinobu et al., 2021). It is unknown how TAM chow compares to TAM injection as regards recombination efficiency and appropriate dosing schedules for adult neurogenesis research.

Here, we compare TAM administration by injection and chow, both in terms of the specificity and efficiency of LoxP recombination and the inherent effects of TAM treatment itself on adult neurogenesis. Using a CreER^T2^ model targeted to NSPCs by a Nestin promoter, our findings suggest that TAM chow can induce LoxP recombination in hippocampal NSPCs with similar specificity as TAM injection, but that TAM chow results in much lower recombination efficiency than injections, likely because of chow avoidance and therefore lower TAM exposure. We also show that both TAM injection and TAM chow paradigms have inherent, although different, effects on adult neurogenesis, underscoring the need to compare TAM-treated experimental animals to genetic controls similarly treated with TAM, regardless of route of administration.

## Materials and Methods

### Mice

All mice were group housed with three to five mice per cage in The Ohio State University Psychology building mouse vivarium in standard ventilated cages on a 12/12 h light/dark cycle (lights on 6:30 A.M.) with *ad libitum* access to food and water. Male and female mice were eight to nine weeks old at the time of the experiment and housed in groups of two to four. All animal use was in accordance with institutional guidelines approved by The Ohio State University Institutional Animal Care and Use Committee.

### Experimental design and statistical analysis

#### Study 1: TAM injection versus TAM chow

Seven- to nine-week-old NestinCreER^T2^ (Lagace et al., 2007; Jackson #016261); R26R-LoxP-STOP-LoxP-EYFP (Srinivas et al., 2001; Jackson #007909) littermates were randomly assigned by whole cage to one of four groups: TAM inject d(day)7 perfuse (*n* = 10), TAM inject d14 perfuse (*n* = 11), TAM chow 10 d (*n* = 7), TAM chow 14 d (*n* = 11). Mice were bred by crossing wild-type C57Bl/6J mice with mice homozygous for both NestinCreER^T2^ and R26R-LoxP-STOP-LoxP-EYFP; thus, offspring were heterozygous for both transgenes. TAM-injected mice received daily 180 mg/kg/d intraperitoneal injections of TAM for 5 d. TAM chow mice were first acclimated to standard chow provision in a dish on the cage floor for 3 d before switching to TAM chow. Food was weighed and refreshed every 2–4 d. At the indicated tissue harvest time, mice were anesthetized with an 87.5 mg/kg ketamine, 12.5 mg/kg xylazine mixture, and then transcardially perfused with ice-cold 0.1 m PBS. Body weight data were analyzed by a repeated measures mixed-effects model (day × TAM) within each sex, followed by Sidak’s multiple comparisons within day. This analysis was performed within sex individually to prevent the strong main effect of sex on body weight from masking any more subtle effects of TAM. Food consumption was analyzed by two-way ANOVA (sex × day) followed by Sidak’s multiple comparisons between days within sex. Cell count data were analyzed by one-way ANOVA followed by Tukey’s *post hoc* tests or by two-way ANOVA (hippocampal subregion × group) followed by Tukey’s *post hoc* multiple comparisons between groups within subregion.

#### Study 2: TAM versus vehicle injection

Wild-type C57Bl6/J mice were obtained at six weeks of age from The Jackson Laboratory (#000664) and allowed to acclimate for two weeks. Cages of mice were randomly assigned to either TAM (*n* = 11 mice) or vehicle (sunflower oil, *n* = 12 mice) injection groups. TAM injections were as described for study 1. On the last 3 d of TAM/vehicle injection, mice also all received daily bromodeoxyuridine (BrdU) injections (150 mg/kg/d, i.p.). Twenty-one days after the last TAM/BrdU injections, mice received a single injection of ethynyldeoxyuridine (EdU; 150 mg/kg, i.p.) and were perfused as in Study 1 2 h later. Body weight data were analyzed by a repeated measures mixed-effects model (day × TAM) within each sex, followed by Sidak’s multiple comparisons within day. BrdU+/EdU+ density was compared by unpaired *t* test. Density and proportion of BrdU/DCX/NeuN or EdU/GFAP/SOX2 cell subtypes was compared by two-way repeated measures ANOVA followed by *post hoc* Sidak’s multiple comparisons within cell type.

#### Study 3: TAM versus vehicle chow

Wild-type C57Bl6/J mice were obtained at six weeks of age from The Jackson Laboratory (#000664) and allowed to acclimate for one week. Cages of mice were randomly assigned to either TAM (*n* = 11 mice) or vehicle (*n* = 12 mice) chow groups. Diet was provided as described for Study 1 for 14 d. On the last 3 d of TAM/vehicle chow, mice also all received daily BrdU injections (150 mg/kg/d, i.p.). 21 d after the last TAM/BrdU injections, mice received a single injection of EdU (150 mg/kg, i.p.) and were perfused as in Study 1 2 h later. Food consumption was analyzed by repeated measures two-way ANOVA (chow type × day) followed by Sidak’s multiple comparisons between chow type within days. Remaining analysis for this experiment is similar to that for Study 2.

### TAM injection preparation

TAM (Fisher #50-115-2413) was dissolved overnight at 20 mg/ml at 37°C in sterile sunflower oil then stored at +4°C for one week maximum, all while protected from light. Cumulative TAM dose for each mouse was calculated by adding the mg of TAM in each injection over the 5 d of the dosing schedule.

### TAM and vehicle chow

TAM chow (500 mg TAM/kg diet, TD.130858, also contains 49.5 g/kg sucrose; 15.4% protein, 55.1% carbohydrate, and 3.4% fat by weight; and 0.25 g/kg of red food color) and matched vehicle chow (15.4% protein, 55.1% carbohydrate, and 3.4% fat by weight; 50 g/kg of sucrose; and 0.25 g/kg of blue food color) were obtained from Envigo and stored at +4°C in the dark until given to mice. Chow was refreshed every 3 d per manufacturer recommendations.

### BrdU and EdU preparation

BrdU (Sigma, #B5002) and EdU (Click Chemistry Tools, #1149-500) were each dissolved fresh in sterile physiological saline at 10 mg/ml.

### Tissue processing

Tissue fixation, slicing, and immunofluorescent staining were performed similarly to our previous work (Dause and Kirby, 2020) using the antibodies described in Table 1. In brief, brains were postfixed in 4% paraformaldehyde (Fisher #AC169650010) in 0.1 m phosphate buffer at +4°C for 24 h then equilibrated in 30% sucrose (Fisher S5-3) in 0.1 m PBS (Fisher #BP399-20) at +4°C before slicing on a freezing microtome (Leica) in a 1:12 series of 40-μm-thick coronal sections. Sections were stored in cryoprotectant medium at −20°C until processed for immunofluorescent staining. Free-floating sections were rinsed in PBS, blocked in 1% normal donkey serum (Jackson ImmunoResearch #017000121), 0.3% Triton X-100 (Fisher #AC215682500) in PBS and incubated in primary antibody in blocking solution overnight at 4°C on rotation. The next day, sections were rinsed, incubated in secondary antibody in blocking solution for 2 h at room temperature on rotation and then rinsed and counterstained with Hoechst 33342 (1:2000 in PBS, Fisher #H3570) before being mounted on SuperFrost Plus slides (Fisher #12-550-15), dried and coverslipped with Prolong Gold Antifade mounting medium (Fisher #P36934). For BrdU staining, sections were processed for other antibodies first then postfixed in 4% paraformaldehyde for 10 min at room temperature. Sections were then rinsed and incubated in 2N HCl (Fisher A144500) at 37°C for 30 min, followed by rinsing, blocking and primary/secondary incubation as described above. For EdU click labeling, sections were click reacted using a Click&Go EdU 488 imaging kit (Click Chemistry Tools, #1324) according to manufacturer instructions before proceeding with subsequent immunolabeling. Slides were all dried overnight at room temperature in the dark and then stored long-term at +4°C. Other materials used for tissue processing, as well as for animal treatment, are in Table 2.

**Table 1:** Antibodies used

Primary antibody	Vendor/product number	RRID	Dilution	Secondary	Vendor/product number	Dilution	RRID
Rabbit anti-GFP	ThermoFisher	AB_221569	1:1000	Donkey Alexa Fluor 488 anti-rabbit	Fisher	1:500	AB_2535792
	A11122				A21206		
Mouse anti-GFAP	EMD Millipore	AB_11212597	1:1000	Donkey Alexa Fluor 647 anti-mouse	Fisher	1:500	AB_162542
	MAB360				A31571		
Rat anti-SOX2	Affymetrix eBioscience	AB_11219471	1:1000	Donkey Alexa Fluor 594 anti-rat	Fisher	1:500	AB_2535795
	14–9811				A21209		
Rabbit anti-DCX	Cell Signaling	AB_561007	1:500	Donkey Alexa Fluor 555 anti-rabbit	Fisher	1:500	AB_162543
	4604				A31572		
Mouse anti-NeuN	EMD Millipore	AB_2298772	1:1000	Donkey Alexa Fluor 647 anti-mouse	Fisher	1:500	AB_162542
	MAB377				A31571		
Rat anti-BrdU	Bio-Rad	AB_609568	1:500	Donkey Alexa Fluor 488 anti-mouse	Fisher	1:500	AB_141607
	OBT0030				A21202		

List of primary and secondary antibodies.

**Table 2:** Other reagents used

Item	Vendor	Product number	RRID or link
NestinCreER^T2^ mice	The Jackson Laboratory	#016261	IMSR_JAX:016261
R26R-LoxP-STOP- LoxP-EYFP	The Jackson Laboratory	#007909	IMSR_JAX:007909
BrdU	Sigma	#B5002	https://www.sigmaaldrich.com/US/en/product/sigma/b5002?gclid=Cj0KCQiAjJOQBhCkARIsAEKMtO2Z8yDVbyW9L9oduQd8T7l-a8P6oFzW-2kFd-oo4Nm87mNJH-mzwZQaAgXJEALw_wcB
EdU	Click Chemistry Tools	#1149-500	https://clickchemistrytools.com/product/5-ethynyl-2%E2%80%B2-deoxyuridine-edu/
TAM chow	Envigo	TD.130858	https://www.envigo.com/tamoxifen-custom-diets
Paraformaldehyde	Fisher	#AC169650010	https://www.fishersci.com/shop/products/paraformaldehyde-90-pure-thermo-scientific/AC169650010
Sucrose	Fisher	S5-3	https://www.fishersci.com/shop/products/sucrose-crystalline-certified-acs-fisher-chemical-3/S53#?keyword=S5-3
PBS	Fisher	#BP399-20	https://www.fishersci.com/shop/products/pbs-phosphate-buffered-saline-10x-solution-fisher-bioreagents/BP39920?searchHijack=true&searchTerm=BP399-20&searchType=RAPID&matchedCatNo=BP399-20
Normal donkey serum	Jackson ImmunoResearch	#017000121	AB_2337258
Triton X-100	Fisher	#AAA16046AE	https://www.fishersci.com/shop/products/triton-x-100-thermo-scientific/AAA16046AE?searchHijack=true&searchTerm=triton-x-100-thermo-scientific&searchType=Rapid&matchedCatNo=AAA16046AE
Hoechst 33342	Fisher	#H3570	https://www.fishersci.com/shop/products/molecular-probes-hoechst-33342-trihydrochloride-trihydrate/H3570#?keyword=H3570
SuperFrost Plus slides	Fisher	#12-550-15	https://www.fishersci.com/shop/products/fisherbrand-superfrost-plus-microscope-slides-2/1255015#?keyword=12-550-15
Prolong Gold Antifade mounting medium	Fisher	#P36934	https://www.fishersci.com/shop/products/molecular-probes-prolong-gold-antifade-mountant-5/P36934?searchHijack=true&searchTerm=P36934&searchType=RAPID&matchedCatNo=P36934
12N HCl	Fisher	A144500	https://www.fishersci.com/shop/products/hydrochloric-acid-certified-acs-plus-fisher-chemical-10/A144500#?keyword=A144500
Click&Go EdU 488 imaging kit	Click Chemistry Tools	#1324	https://clickchemistrytools.com/product/click-go-edu-488-imaging-kit/

List of other major reagents.

### Cell quantification

Similar to our previous work (Dause and Kirby, 2020), the dentate gyrus of the hippocampus was imaged in 15-μm z-stacks at 20× magnification using a Zeiss Axio Observer Z.1 with apotome digital imaging system and Axiocam 506 monochrome camera (Zeiss). EYFP+ cells were identified based on EYFP+ cytoplasm around a Hoechst+ nucleus. EYFP+ cells were considered radial glia-like neural stem cells (RGL-NSCs) if they had a SOX2+ nucleus in the subgranular zone and colocalized with GFAP in the cytoplasm with an apical morphology. They were considered intermediate progenitor cells (IPCs) if they had a SOX2+ nucleus in the subgranular zone but did not have a GFAP+ cytoplasm. They were considered astrocytes if they had a SOX2+ nucleus and colocalized with GFAP in the cytoplasm with a stellate morphology. They were considered immature neurons/neuroblasts if they had a Hoechst+ nucleus in the sungranular zone and a cytoplasm that co-localized with DCX. BrdU+ cells were counted in the subgranular zone and granule cell layer throughout the dentate gyrus. Colabeling with DCX and NeuN was assessed in each BrdU+ cell in the z-stacks as surrounding DCX+ cytoplasm and/or nuclear NeuN overlap with BrdU. All cell counts were divided by dentate gyrus area sampled to yield a density of cells.

### Software

Statistical analyses were performed in GraphPad Prism (version 9.3.1). Graphical abstract was made using BioRender software.

## Results

### TAM chow results in weight loss and less total TAM exposure compared with TAM injection

**Figure 1.**
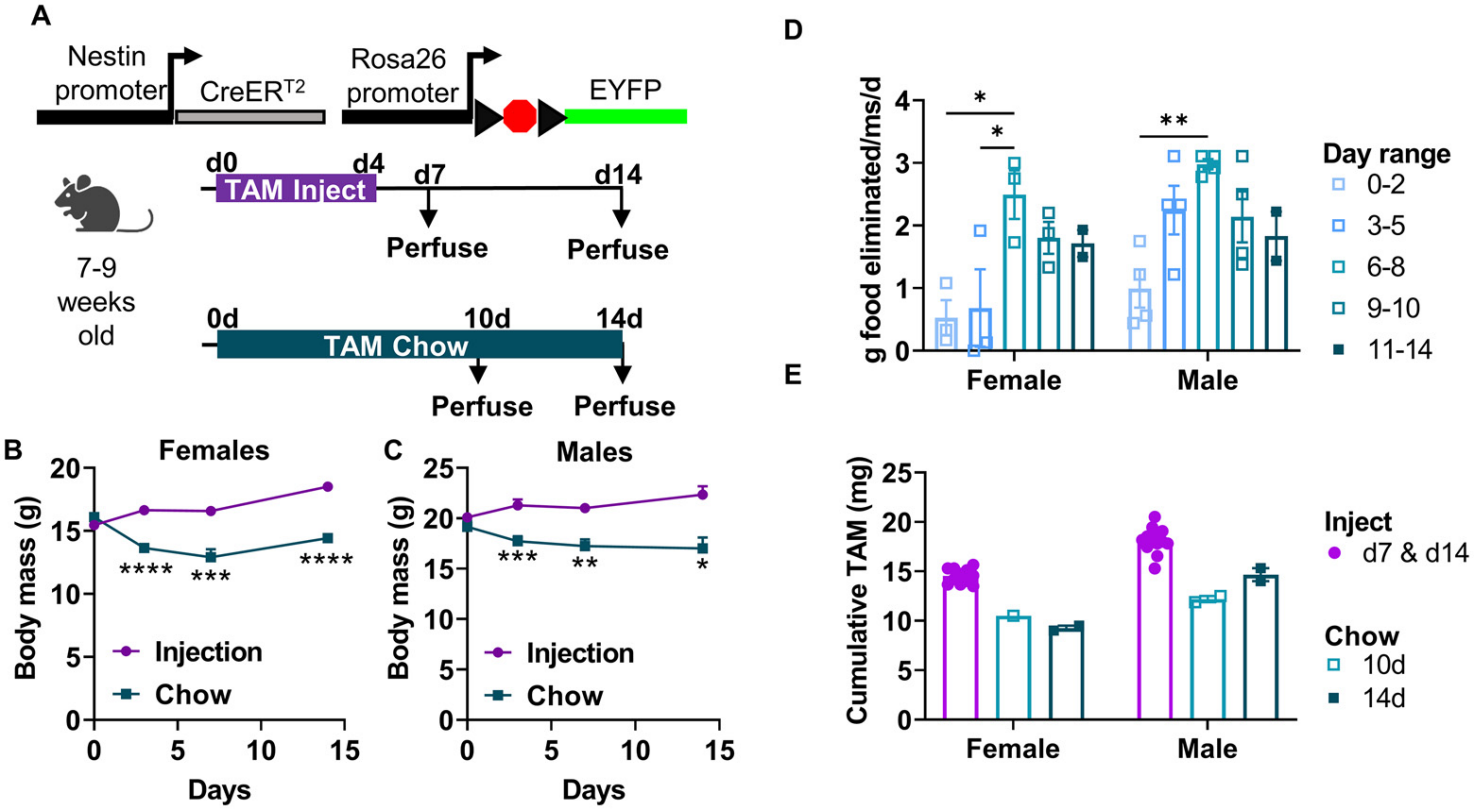
TAM chow causes food avoidance and results in less TAM exposure compared with TAM injection. ***A***, Experimental model and timeline. Adult Nestin-CreER^T2^+/−;Rosa26-LoxP-STOP-LoxP-EYFP+/− mice were either injected with TAM (intraperitoneally) or voluntarily fed TAM chow. ***B***, ***C***, Body mass (in grams) of female (day: *F*_(1.637,24.56)_ = 8.314, *p *=* *0.0029; TAM: *F*_(1,20)_ = 45.35, *p < *0.0001; day × TAM interaction: *F*_(3,45)_ = 23.42, *p *<* *0.0001; ***B***) and male (day: *F*_(0.5896,9.630)_ = 1.338, *p *=* *0.2412; TAM: *F*_(1,23)_ = 21.96, *p = *0.0001; day × TAM interaction: *F*_(3,49)_ = 10.40, *p *<* *0.0001; ***C***) mice during and after TAM treatment. Mean ± SEM of *n* = 4–11 mice/time point. ***D***, TAM chow consumption estimation (g of food not recovered to weigh) per mouse (ms) per day, averaged within cage (sex: *F*_(1,22)_ = 6.139, *p *=* *0.0214; day: *F*_(4,22)_ = 8.310, *p *=* *0.0003; interaction: *F*_(4,22)_ = 1.159, *p *=* *0.3558). Mean ± SEM of *n* = 2–4 cages shown. ***E***, Estimated cumulative TAM exposure (milligram (mg) of TAM) in injected and chow-fed mice (sex: *F*_(1,25)_ = 39.49, *p *<* *0.0001; TAM: *F*_(2,25)_ = 51.35, *p *<* *0.0001; interaction: *F*_(2,25)_ = 2.626, *p *=* *0.0922). Mean ± SEM shown from *n* = 11–13 mice (injection) or *n* = 1–2 cages (chow). *p <* *0.05, **p <* *0.01, ***p <* *0.001, ****p <* *0.0001, as determined by Sidak’s multiple comparisons test following a mixed-effects analysis (***B***, ***C***) or two-way ANOVA (***D***, ***E***).

To compare TAM-induced LoxP recombination in NSPCs between injection and chow-fed methods, we crossed NestinCreER^T2^ mice (Lagace et al., 2007) with Rosa26-LoxP-STOP-LoxP-EYFP mice (Srinivas et al., 2001). At seven to nine weeks of age, NestinCreER^T2^+/−;Rosa26-LoxP-STOP-LoxP-EYFP+/− male and female mice were assigned randomly to either injection or chow groups. Injected mice received a standard 5-d injection regimen and were perfused on days 7 or 14 after the start of injections (Fig. 1*A*). TAM chow-fed mice were given free access to TAM chow for 10 or 14 d before perfusion (Fig. 1*A*). Both male and female TAM chow-fed mice showed lower body mass compared with TAM-injected mice (Fig. 1*B*,*C*). TAM chow-fed mice consumed very little of the chow for the first ∼5 d (Fig. 1*D*), a time period which coincided with the greatest divergence in weight in these mice compared with TAM injected mice. Mice appeared active and well-groomed throughout the experiment. By days 6–8, male and female mice significantly increased their apparent chow consumption, coinciding with stabilization of their body weights. Cumulative TAM exposure was 1.23-fold to 1.57-fold higher in injected mice than chow-fed mice (Fig. 1*E*). It should be noted that some chow was crumbled on the cage floor and could not be weighed. These estimates for chow consumption are most likely overestimates. Nonetheless, these data suggest that mice find TAM chow aversive, resulting in less TAM exposure over 14 d than from 5 d of standard injections.

### TAM chow results in lower recombination efficiency then TAM injections

**Figure 2.**
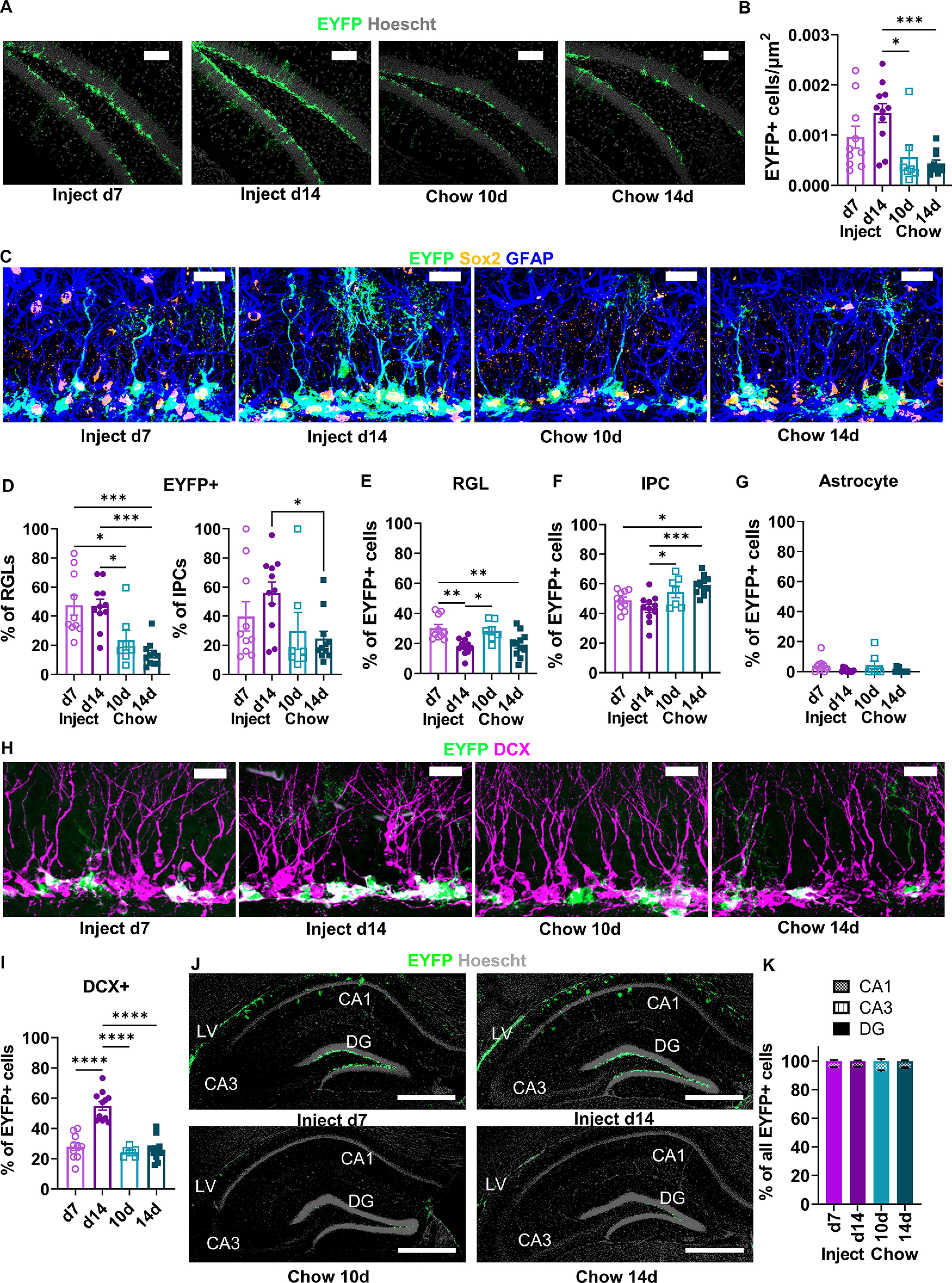
Recombination efficiency is higher following TAM injection than TAM diet. ***A***, Representative images of EYFP+ labeling in the DG. Scale bar: 100 μm. ***B***, Density of EYFP+ cells (cells/μm^2^) in the DG (ordinary one-way ANOVA, *F*_(3,35)_ = 6.885, *p *=* *0.0009). Mean ± SEM of *n* = 7–11 mice. ***C***, Representative images of EYFP, SOX2, and GFAP labeling in the dentate gyrus. Scale bar: 20 μm. ***D***, The percent of phenotypic GFAP+/SOX2+ RGL-NSCs and GFAP-/SOX2+ IPCs that were EYFP+ (RGLs: ordinary one-way ANOVA, *F*_(3,35)_ = 11.43, *p *<* *0.0001; IPCs: ordinary one-way ANOVA, *F*_(3,35)_ = 2.788, *p *=* *0.0550). Mean ± SEM of *n* = 7–11 mice. ***E***, The percent of EYFP+ cells that showed RGL-NSC phenotype (ordinary one-way ANOVA, *F*_(3,35)_ = 7.531, *p *=* *0.0005). Mean ± SEM of *n* = 7–11 mice. ***F***, The percent of EYFP+ cells that showed IPC phenotype (ordinary one-way ANOVA, *F*_(3,35)_ = 7.799, *p *=* *0.0004). Mean ± SEM of *n* = 7–11 mice. ***G***, The percent of EYFP+ cells that showed GFAP+ stellar astrocyte phenotype (ordinary one-way ANOVA, *F*_(3,35)_ = 2.465, *p *=* *0.0784). Mean ± SEM of *n* = 7–11 mice. ***H***, Representative images of EYFP and DCX labeling in the dentate gyrus. Scale bar: 20 μm. ***I***, The percent of EYFP+ cells that were DCX+ (ordinary one-way ANOVA, *F*_(3,35)_ = 34.97, *p *<* *0.0001). Mean ± SEM of *n* = 7–11 mice. ***J***, Representative images of EYFP labeling throughout the hippocampus. DG = dentate gyrus. Scale bar: 500 μm. ***K***, Percent of hippocampal EYFP+ cells found in the CA1, CA3, and dentate gyrus (two-way repeated measures ANOVA; TAM × area: *F*_(6,70)_ = 3.338, *p *=* *0.0060; TAM: *F*_(3,35)_ = 1.051, *p *=* *0.3822; area: *F*_(1.186,41.50)_ = 20 157, *p *<* *0.0001; subject: *F*_(35,70)_ = 6.492e-007, *p *>* *0.9999). Mean ± SEM of *n* = 7–11 mice. *p <* *0.05, **p <* *0.01, ***p <* *0.001, ****p <* *0.0001, as determined by Tukey’s multiple comparisons test.

**Figure 3.**
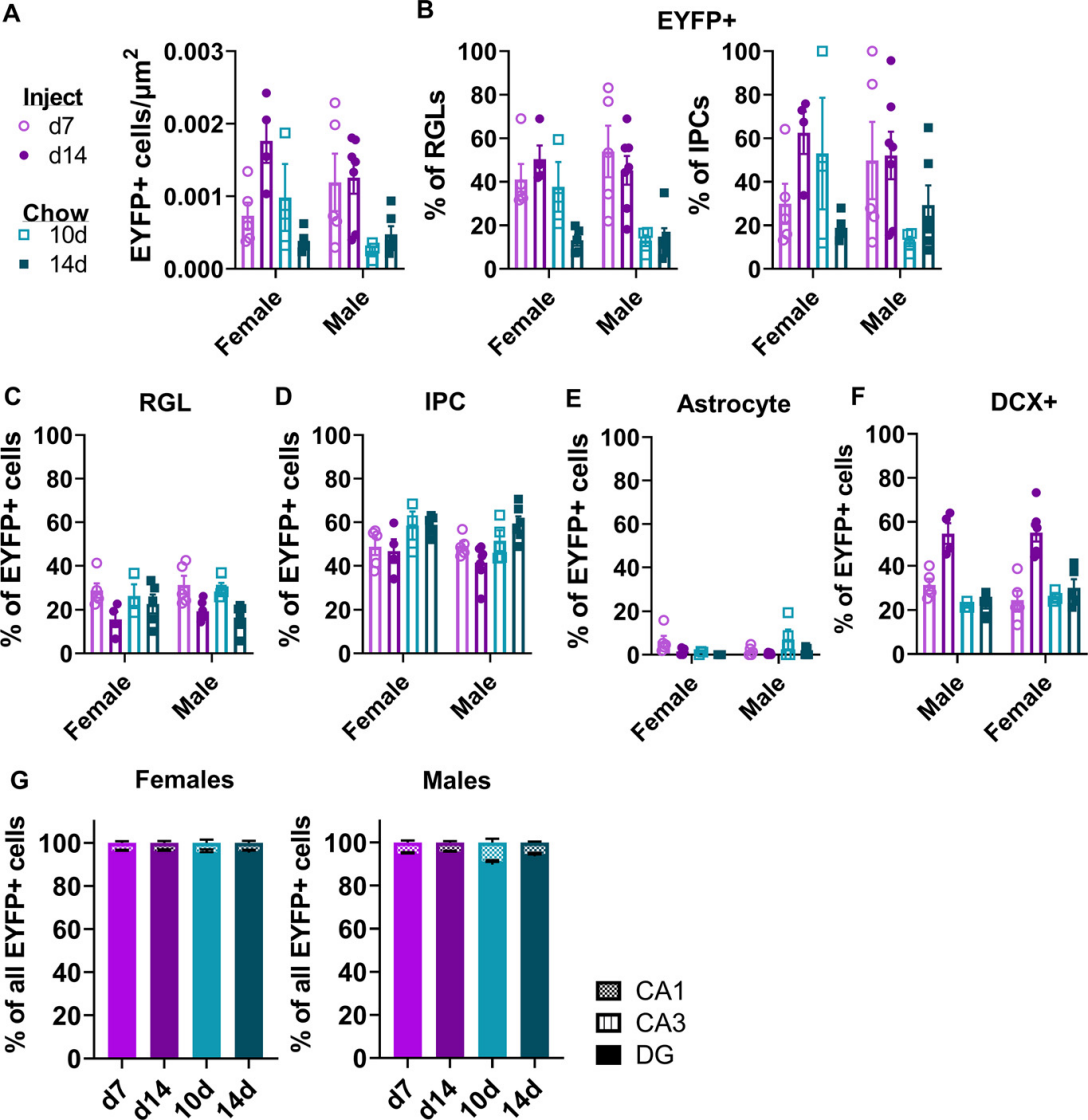
Recombination efficiency and labeled cell types do not depend on sex. ***A***, Density of EYFP+ cells (cell/μm^2^) in the dentate gyrus by sex (two-way ANOVA; interaction: *F*_(3,31)_ = 2.335, *p *=* *0.0931; sex: *F*_(1,31)_ = 0.9785, *p *=* *0.3302; TAM: *F*_(3,31)_ = 8.017, *p *=* *0.0004). Mean ± SEM of *n* = 3–7 mice. ***B***, The percent of phenotypic GFAP+/SOX2+ RGL-NSCs (two-way ANOVA; interaction: *F*_(3,31)_ = 2.003, *p *=* *0.1341; sex: *F*_(1,31)_ = 0.5780, *p *=* *0.4528; TAM: *F*_(3,31)_ = 11.87, *p *<* *0.0001) and GFAP-/SOX2+ IPCs (two-way ANOVA; interaction: *F*_(3,31)_ = 2.152, *p *=* *0.1138; sex: *F*_(1,31)_ = 0.3609, *p *=* *0.5524; TAM: *F*_(3,31)_ = 3.040, *p *=* *0.0436) that were EYFP+, with sex on the horizontal axis. Mean ± SEM of *n* = 3–7 mice. ***C***, The percent of EYFP+ cells that showed RGL-NSC phenotype, analyzed separately by sex (two-way ANOVA; interaction: *F*_(3,31)_ = 1.147, *p *=* *0.3457; sex: *F*_(1,31)_ = 0.1478, *p *=* *0.7032; TAM: *F*_(3,31)_ = 7.335, *p *=* *0.0007). Mean ± SEM of *n* = 3–7 mice. ***D***, The percent of EYFP+ cells that showed IPC phenotype, analyzed separately by sex (two-way ANOVA; interaction: *F*_(3,31)_ = 0.4952, *p *=* *0.6883; sex: *F*_(1,31)_ = 1.078, *p *=* *0.3072; TAM: *F*_(3,31)_ = 6.650, *p *=* *0.0013). Mean ± SEM of *n* = 3–7 mice. ***E***, The percent of EYFP+ cells that showed GFAP+ stellar astrocyte phenotype, analyzed separately by sex (two-way ANOVA; interaction: *F*_(3,31)_ = 2.982, *p *=* *0.0464; sex: *F*_(1,31)_ = 0.1198, *p *=* *0.7316; TAM: *F*_(3,31)_ = 2.411, *p *=* *0.0857). Mean ± SEM of *n* = 3–7 mice. ***F***, The percent of EYFP+ cells that showed DCX+ immature neuron phenotype, analyzed separately by sex (two-way ANOVA; interaction: *F*_(3,31)_ = 1.580, *p *=* *0.2141; sex: *F*_(1,31)_ = 0.2172, *p *=* *0.6444; TAM: *F*_(3,31)_ = 33.62, *p *<* *0.0001). Mean ± SEM of *n* = 3–7 mice. ***G***, Percent of hippocampal EYFP+ cells found in the CA1, CA3, and dentate gyrus (DG) in male (two-way repeated measures ANOVA; group × region: *F*_(6,36)_ = 6.259, *p *=* *0.0001; group: *F*_(3,18)_ = 1.636, *p *=* *0.2162; region: *F*_(1.303,23.46)_ = 14,034, *p *<* *0.0001; subject: *F*_(18,36)_ = 3.413e-007, *p *>* *0.9999) and female (two-way repeated measures ANOVA; group × region: *F*_(6,26)_ = 0.2057, *p *=* *0.9719; group: *F*_(3,13)_ = 1.090, *p *=* *0.3880; region: *F*_(1.193,15.51)_ = 12,616, *p *<* *0.0001; subject: *F*_(13,26)_ = 1.595e-006, *p *>* *0.9999) mice at d7 and d14 dpi and 10 and 14 d after TAM chow initiation. Mean ± SEM of *n* = 3–5 female mice and 4–7 male mice. *p <* *0.05, **p <* *0.01, ***p <* *0.001, ****p <* *0.0001, as determined by Sidak’s multiple comparisons test (***A–F***) or Tukey’s multiple comparisons test (***G***).

We next compared EYFP+ cell density in the subgranular zone of the DG to determine the relative efficacy of TAM-induced Rosa26-LoxP-STOP-LoxP-EYFP recombination in these mice. As expected based on previous studies with this line of NestinCreER^T2^ mice (Lagace et al., 2007; Sun et al., 2014; Dause and Kirby, 2020), EYFP expression filled cell bodies and was strongly localized to the subgranular zone in both TAM-injected and TAM chow-fed mice (Fig. 2*A*). 10- and 14-d TAM chow-fed mice had 2.54-fold and 3.31-fold fewer EYFP+ cells compared with injected mice perfused 14 d after the start of injections (Fig. 2*A*,*B*; Extended Data Fig. 2-1). We next quantified the efficiency of EYFP expression specifically in GFAP+/SOX2+ RGL-NSCs and GFAP-/SOX2+ IPCs (Fig. 2*C*). TAM-injected mice showed EYFP expression in slightly less than half of RGL-NSCs (47.55 ± 6.34% and 47.18 ± 4.63% at 7 and 14 d after injection start) while TAM chow-fed mice showed EYFP expression in less than a quarter of RGL-NSCs (23.61 ± 6.72% and 13.80 ± 2.53% at 10 and 14 d of chow; Fig. 2*D*). Among IPCs, TAM-injected mice showed EYFP expression in 39.86 ± 10.00% and 55.91 ± 7.65% of IPCs at 7 and 14 d after injection start. TAM chow-fed mice again showed significantly less EYFP expression among IPCs (29.89 ± 12.72% and 24.55 ± 5.15% at 10 and 14 d of chow; Fig. 2*D*). Separate analysis of males and females yielded similar results in each sex (Fig. 3*A*,*B*). Together, these findings suggest lower, but detectable, LoxP-recombination following 10–14 d of TAM chow feeding compared with a standard 5-d injection regimen.

10.1523/ENEURO.0422-21.2022.f2-1 Figure 2-1 Raw data supporting Figure 2. Download Figure 2-1, XLSX file.

### TAM chow and TAM injections show similar recombination specificity

We next quantified the cell phenotype of EYFP+ cells in the DG. At day 7 after TAM injection start, 30.11 ± 2.49% of DG EYFP+ cells were phenotypic RGL-NSCs (Fig. 2*E*). Mice fed TAM chow for 10 d showed a similar fraction of EYFP+ cells that were phenotypic RGL-NSCs (28.48 ± 2.48%). By 14 d after TAM injection start, the percent of EYFP+ cells that were RGL-NSCs dropped significantly compared with 7 d to 17.95 ± 1.61%. This decrease in proportion of EYFP+ cells that are RGL-NSCs is consistent with differentiation of the recombined, EYFP+ population over time. In the 14-d chow group, 19.28 ± 2.47% of EYFP+ cells were phenotypic RGL-NSCs, a fraction that was slightly but significantly lower than the 7-d injection group. The percent of EYFP+ cells showing IPC phenotype were similar at 7 and 14 d after injection start (48.99 ± 2.73% and 43.44 ± 2.73%; Fig. 2*F*). However, TAM chow-fed mice showed slightly, although significantly, higher percentages of EYFP+ cells that were phenotypic IPCs than TAM-injected mice (10 d: 54.54 ± 3.82%; 14 d: 59.17 ± 1.91%). As expected, very few EYFP+ cells showed an astrocytic phenotype in all groups (d7 inject: 4.00 ± 1.45%; d14 inject: 0.83 ± 0.36%; 10 d chow: 4.40 ± 2.76%; 14 d chow: 0.61 ± 1.38%; Fig. 2*G*). We also used co-labeling of doublecortin (DCX) with EYFP to quantify the portion of EYFP+ cells that were phenotypic neuroblasts/immature neurons. DCX co-labeling increased significantly from 7 to 14 d after TAM injection start (28.00 ± 2.61% to 54.91 ± 2.90% of EYFP+ cells co-labeled for DCX), again consistent with the maturation of the recombined, EYFP+ cell population over time. Both TAM chow-fed groups showed similar percentages of EYFP+ cells co-labeling for DCX as the 7-d injection group (10 d: 24.44 ± 1.05%; 14 d: 26.07 ± 2.29%). Separate analysis of males and females yielded similar results in each sex (Fig. 3*C–F*).

To assess ectopic recombination in non-neurogenic regions of the hippocampus, we quantified EYFP+ cells in CA1 and CA3. In all groups, EYFP+ cells were detectable in CA1 and CA3 but the rates of EYFP expression were low, with >92% of all EYFP+ cells being located in the dentate gyrus (Fig. 2*J–K*). Groups did not significantly differ from each other in EYFP localization to the dentate gyrus and separate analysis of males and females yielded similar results in each sex (Fig. 3*G*). All together, these data suggest that both TAM injection and TAM chow drive recombination predominantly in NSPCs with no notable differences in their specificity. They also suggest that 10–14 d of chow exposure leads to a recombined population that is most phenotypically similar to that found 7 d after TAM injection start.

### TAM injection leads to a long-term suppression of cell proliferation

**Figure 4.**
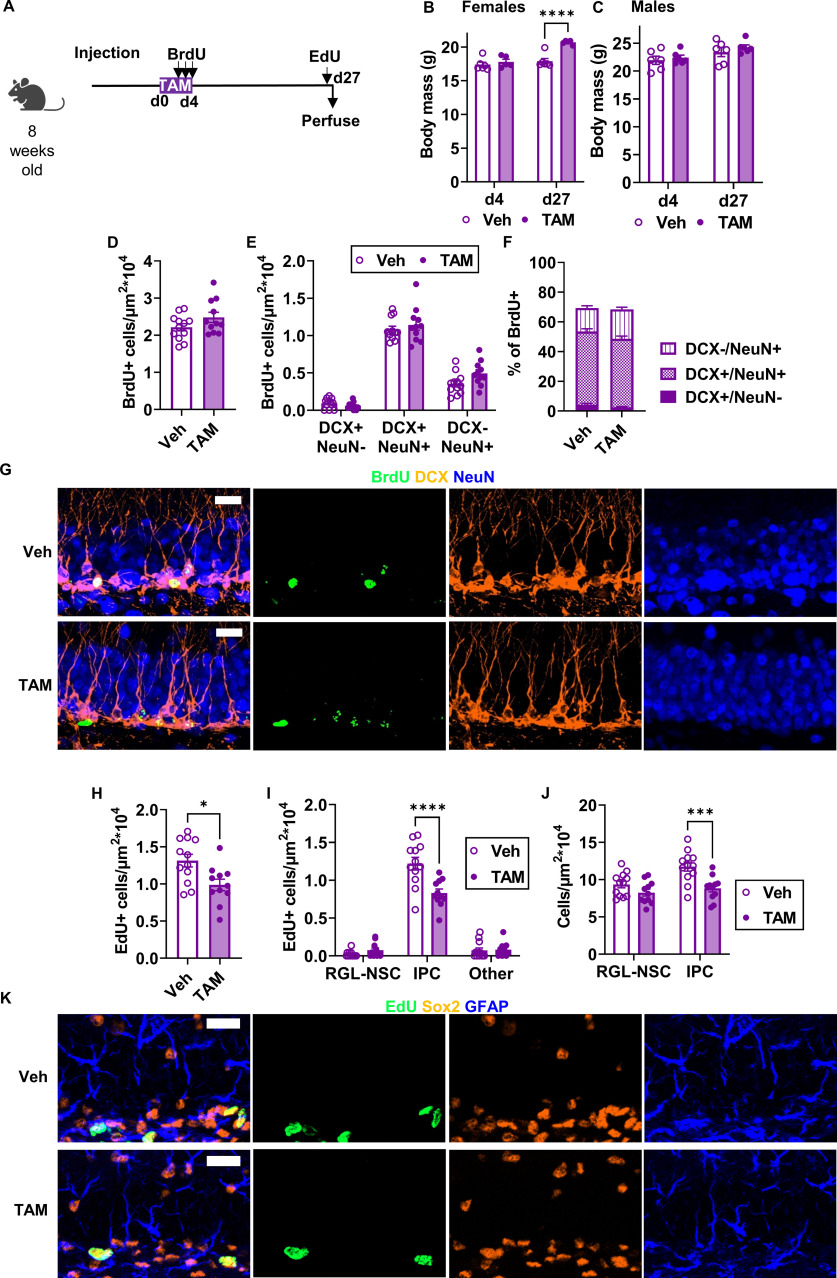
TAM injection suppresses progenitor cell proliferation three weeks after TAM. ***A***, Experimental model and timeline. Adult wild-type mice were injected with TAM daily for 5 d then perfused three weeks later. Mice received three daily BrdU injections on the last 3 d of TAM and one EdU injection 2 h before perfusion. ***B***, ***C***, Average body mass of females (two-way repeated measures ANOVA; day × TAM: *F*_(1,9)_ = 11.17, *p *=* *0.0086; day: *F*_(1,9)_ = 22.53, *p *=* *0.0010; TAM: *F*_(1,9)_ = 21.55, *p *=* *0.0012; subject: *F*_(9,9)_ = 0.9448, *p *=* *0.5330) and males (two-way repeated measures ANOVA; day × TAM: *F*_(1,10)_ = 0.3575, *p *=* *0.5632; day: *F*_(1,10)_ = 24.83, *p *=* *0.0006; TAM: *F*_(1,10)_ = 0.6152, *p *=* *0.4510; subject: *F*_(10,10)_ = 6.138, *p *=* *0.0042) at the end of TAM treatment (d4) and three weeks later (d27). Mean ± SEM of *n* = 5–6 female and 6 male mice. ***D***, Density of BrdU+ cells (cells/μm^2^ × 10^4^) in the dentate gyrus (unpaired *t* test, *t*_(21)_ = 1.613, *p *=* *0.1217). Mean ± SEM of *n* = 11–12 mice. ***E***, Density of dentate gyrus BrdU+ cells co-labeled with DCX and/or NeuN (per μm^2^ × 10^4^; two-way repeated measures ANOVA; cell type × TAM: *F*_(2,42)_ = 2.461, *p *=* *0.0976; cell type: *F*_(2,42)_ = 371.0, *p *<* *0.0001; TAM: *F*_(1,21)_ = 1.843, *p *=* *0.1890; subject: *F*_(21,42)_ = 1.698, *p *=* *0.0714). Mean ± SEM of *n* = 11–12 mice. ***F***, Percent of BrdU+ dentate gyrus cells co-labeled for DCX and/or NeuN (two-way repeated measures ANOVA; cell type × TAM: *F*_(2,42)_ = 3.027, *p *=* *0.0591; TAM: *F*_(1,21)_ = 0.1108, *p *=* *0.7425; cell type: *F*_(1.606,33.73)_ = 440.6, *p *<* *0.0001; subject: *F*_(21,42)_ = 0.5522, *p *=* *0.9275). Mean ± SEM of *n* = 11–12 mice. ***G***, Representative images of BrdU, DCX, and NeuN labeling. Scale bar: 20 μm. ***H***, Density of EdU+ cells (cells/μm^2^ × 10^4^) in the dentate gyrus (unpaired *t* test, *t*_(21)_ = 2.814, *p *=* *0.0104). Mean ± SEM of *n* = 11–12 mice. ***I***, Density (cells/μm^2^ × 10^4^) of EdU+ phenotypic RGL-NSCs, IPCs, and other cells in the dentate gyrus (two-way ANOVA; interaction: *F*_(2,63)_ = 13.82, *p *<* *0.0001; cell type: *F*_(2,63)_ = 287.1, *p *<* *0.0001; TAM: *F*_(1,63)_ = 8.275, *p *=* *0.0055). Mean ± SEM of *n* = 11–12 mice. ***J***, Density (cells/μm^2^ × 10^4^) of RGL-NSCs and IPCs in the dentate gyrus (two-way repeated measures ANOVA; cell type × TAM: *F*_(1,21)_ = 3.203, *p *=* *0.0879; cell type: *F*_(1,21)_ = 8.683, *p *=* *0.0077; TAM: *F*_(1,21)_ = 15.23, *p *=* *0.0008; subject: *F*_(21,21)_ = 1.029, *p *=* *0.4741). Mean ± SEM of *n* = 11–12 mice. ***K***, Representative images of EdU, SOX2, and GFAP labeling. Scale bar: 20 μm. *p <* *0.05, **p <* *0.01, ***p <* *0.001, ****p <* *0.0001, as determined by Sidak’s multiple comparisons test.

We next asked whether TAM exposure itself disrupts physiological adult hippocampal neurogenesis. Adult wild-type C57BL6/J mice were injected with TAM or matched dose (ml/kg) of vehicle (oil) for 5 d (Fig. 4*A*). Mice received daily injections of BrdU to label dividing cells coincident with the last 3 d of TAM. Mice were perfused 27 d after the first TAM injection (21 d after the last TAM/BrdU injections), 2 h after a single injection of EdU to label acutely proliferating cells. Body weights of female and male mice were not altered by 5 d TAM injection compared with vehicle injection (female: 17.38 ± 0.36 g veh vs 17.80 ± 0.38 g TAM; male: 21.93 ± 0.72 g veh vs 22.38 ± 0.46 g TAM; Fig. 4*B*,*C*). However, after a 21-d recovery from TAM/vehicle injections, TAM-injected females weighed 1.16-fold more than vehicle-injected females (17.88 ± 0.41 g veh vs 20.68 ± 0.13 g TAM; Fig. 4*B*). TAM and vehicle-injected body weights did not significantly differ among male mice after a 21-d recovery (23.40 ± 0.81 g veh vs 24.25 ± 0.45 g TAM; Fig. 4*C*).

**Figure 5.**
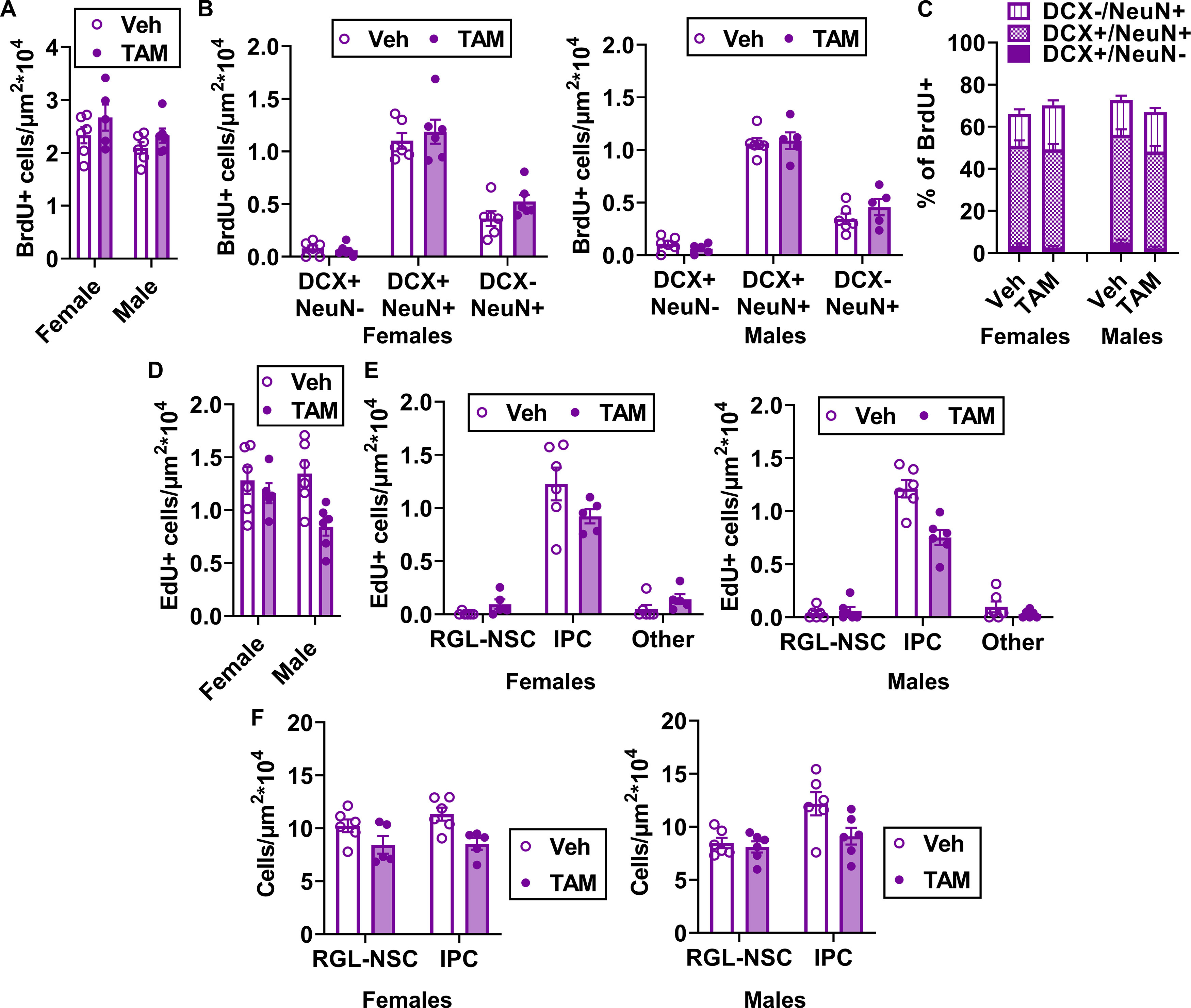
TAM injection effects on cell survival and proliferation do not differ by sex. ***A***, Density of BrdU+ cells (cells/μm^2^ × 10^4^) in the dentate gyrus (two-way ANOVA; interaction: *F*_(1,19)_ = 0.09813, *p *=* *0.7575; sex: *F*_(1,19)_ = 3.232, *p *=* *0.0881; TAM: *F*_(1,19)_ = 3.076, *p *=* *0.0956). Mean ± SEM of *n* = 5–6 mice. ***B***, Density of dentate gyrus BrdU+ cells (cells/μm^2^ × 10^4^) co-labeled with DCX and/or NeuN (three-way repeated measures ANOVA; cell type: *F*_(1.823,34.63)_ = 343.0, *p *<* *0.0001; sex: *F*_(1,19)_ = 0.5866, *p *=* *0.4531; TAM: *F*_(1,19)_ = 1.581, *p *=* *0.2238; cell type × sex: *F*_(2,38)_ = 0.5649, *p *=* *0.5731; cell type × TAM: *F*_(2,38)_ = 2.197, *p *=* *0.1250; sex × TAM: *F*_(1,19)_ = 0.2991, *p *=* *0.5908; cell type × sex × TAM: *F*_(2,38)_ = 0.02294, *p *=* *0.9773). Mean ± SEM of *n* = 6 female mice and 5–6 male mice. ***C***, Percent of BrdU+ dentate gyrus cells labeled for DCX and/or NeuN (three-way repeated measures ANOVA; cell type: *F*_(1.608,30.55)_ = 401.7, *p *<* *0.0001; sex: *F*_(1,19)_ = 0.4138, *p *=* *0.5277; TAM: *F*_(1,19)_ = 0.08557, *p *=* *0.7731; cell type × sex: *F*_(2,38)_ = 0.1847, *p *=* *0.8321; cell type × TAM: *F*_(2,38)_ = 2.828, *p *=* *0.0716; sex × TAM: *F*_(1,19)_ = 3.418, *p *=* *0.0801; cell type × sex × TAM: *F*_(2,38)_ = 0.04,942), *p *=* *0.9518. Mean ± SEM of *n* = 5–6 mice. ***D***, Density of EdU+ cells (cells/μm^2^ × 10^4^) in the dentate gyrus (two-way ANOVA; interaction: *F*_(1,19)_ = 2.991, *p *=* *0.0999; sex: *F*_(1,19)_ = 1.313, *p *=* *0.2660; TAM: *F*_(1,19)_ = 7.928, *p *=* *0.0110). Mean ± SEM of *n* = 5–6 mice. ***E***, Density (cells/μm^2^ × 10^4^) of dentate gyrus EdU+ phenotypic RGL-NSCs, IPCs, and other cells (three-way repeated measures ANOVA; cell type: *F*_(2,38)_ = 274.4, *p *<* *0.0001; sex: *F*_(1,19)_ = 1.313, *p *=* *0.2660; TAM: *F*_(1,19)_ = 7.928, *p *=* *0.0110; cell type × sex: *F*_(2,38)_ = 0.4635, *p *=* *0.6326; cell type × TAM: *F*_(2,38)_ = 12.86, *p *<* *0.0001; sex × TAM: *F*_(1,19)_ = 2.991, *p *=* *0.0999; cell type × sex × TAM: *F*_(2,38)_ = 0.1659, *p *=* *0.8477). Mean ± SEM of *n* = 5–6 female mice and 6 male mice. ***F***, Density (cells/μm^2^ × 10^4^) of RGL-NSCs and IPCs in the dentate gyrus, by sex (three-way repeated measures ANOVA; cell type: *F*_(1,19)_ = 9.308, *p *=* *0.0066; sex: *F*_(1,19)_ = 0.1083, *p *=* *0.7457; TAM: *F*_(1,19)_ = 14.12, *p *=* *0.0013; cell type × sex: *F*_(1,19)_ = 3.346, *p *=* *0.0831; cell type × TAM: *F*_(1,19)_ = 3.699, *p *=* *0.0696; sex × TAM: *F*_(1,19)_ = 0.3142, *p *=* *0.5817; cell type × sex × TAM: *F*_(1,19)_ = 0.7541, *p *=* *0.3960). Mean ± SEM of *n* = 5–6 female mice and 6 male mice. *p <* *0.05, **p <* *0.01, ***p <* *0.001, ****p <* *0.0001, as determined by Sidak’s multiple comparisons test.

The total density of surviving BrdU+ cells did not differ between TAM and vehicle-injected mice (2.48 ± 0.14 × 10^−4^ vs 2.22 ± 0.10 × 10^−4^ BrdU+ cells/μm^2^, respectively; Fig. 4*D*; Extended Data Fig. 4-1). The total density of BrdU+ cells expressing neuronal phenotypic markers DCX and/or NeuN also did not differ between TAM and vehicle-injected mice (Fig. 4*E*,*G*). Almost half of BrdU+ cells in both groups co-expressed both DCX and NeuN (49.36 ± 1.84% veh and 46.24 ± 1.75% TAM) while slightly less than a fifth of BrdU+ cells in both groups were single positive for the mature neuronal marker NeuN (15.82 ± 1.47% veh and 19.78 ± 1.43% TAM; Fig. 4*F*,*G*). A small proportion of BrdU+ cells were single positive for DCX (4.12 ± 0.91% veh and 2.43 ± 0.55% TAM). Altogether, ∼70% of BrdU cells showed a neuronal phenotype via DCX and/or NeuN co-labeling (69.38 ± 2.16% veh and 68.45 ± 1.71% TAM). Similar results were found when males and females were analyzed separately (Fig. 5*A–C*). Combined, these results suggest that TAM injection during BrdU-labeling does not alter the density of new-born neurons three weeks later.

10.1523/ENEURO.0422-21.2022.f4-1 Figure 4-1 Raw data supporting Figure 4. Download Figure 4-1, XLSX file.

We also quantified cell proliferation of RGL-NSCs and IPCs three weeks after TAM using EdU to label proliferating cells. TAM injection led to a 1.32-fold suppression of total EdU+ cell density in the dentate gyrus compared with vehicle-injected mice (1.31 ± 0.09 × 10^−4^ cells/μm^2^ veh vs 0.99 ± 0.08 × 10^−4^ cells/μm^2^ TAM; Fig. 4*H*,*K*). Classification of EdU+ cells as RGL-NSCs, IPCs or neither revealed that this reduction in EdU labeling was driven primarily by loss of EdU+ IPCs (Fig. 4*I*,*K*). Quantification of total RGL-NSCs and IPCs similarly showed a significant loss of total IPC density in TAM-treated mice, with a more moderate, nonsignificant decrease in RGL-NSC density (Fig. 4*J*,*K*). Similar results were found when males and females were analyzed separately (Fig. 5*D–F*). These findings suggest that TAM injection causes a long-term suppression of IPC proliferation that is evident three weeks after TAM has ended.

### TAM chow enhances adult neurogenesis acutely but does not suppress cell proliferation long term

**Figure 6.**
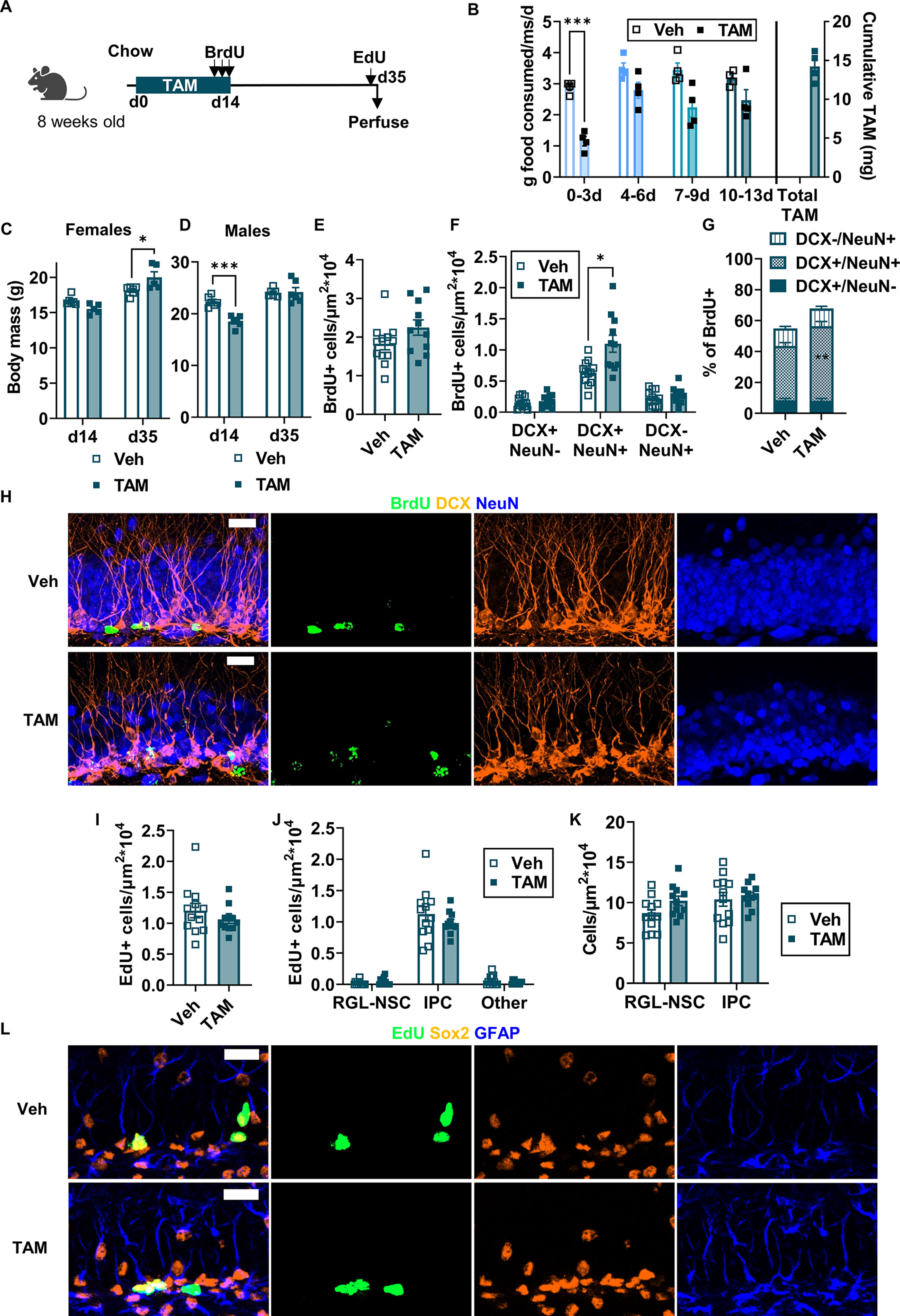
TAM chow alters the phenotype of surviving cells 35 d after initial exposure. ***A***, Experimental model and timeline. Mice were injected with BrdU in the last 3 d of the 14-d TAM chow period. EdU was injected 35 d after initial chow exposure. ***B***, Grams of food consumed per mouse (ms) per day over each 3- to 4-d period of diet exposure and TAM consumed in milligrams (mg) (two-way repeated measures ANOVA; day × TAM: *F*_(3,18)_ = 2.322, *p *=* *0.1095; day: *F*_(1.564,9.384)_ = 8.978, *p *=* *0.0088; TAM: *F*_(1,6)_ = 45.86, *p *=* *0.0005; subject: *F*_(6,18)_ = 0.9896, *p *=* *0.4613). Mean ± SEM of *n* = 4 mice. ***C***, In female mice, body mass in grams 14 and 35 d after diet initiation (two-way repeated measures ANOVA; day × TAM: *F*_(1,9)_ = 10.62, *p *=* *0.0099; day: *F*_(1,9)_ = 42.05, *p *=* *0.0001; TAM: *F*_(1,9)_ = 0.8454, *p *=* *0.3818; subject: *F*_(9,9)_ = 0.7591, *p *=* *0.6560). Mean ± SEM of *n* = 5–6 mice. ***D***, In male mice, body mass in grams 14 and 35 d after diet initiation (two-way repeated measures ANOVA; day × TAM: *F*_(1,10)_ = 18.18, *p *=* *0.0017; day: *F*_(1,10)_ = 62.45, *p *<* *0.0001; TAM: *F*_(1,10)_ = 8.540, *p *=* *0.0152; subject: *F*_(10,10)_ = 1.886, *p *=* *0.1658). Mean ± SEM of *n* = 6 mice. ***E***, Density of BrdU+ cells (cells/μm^2^ × 10^4^) in the dentate gyrus (unpaired *t* test, *t*_(21)_ = 1.675, *p *=* *0.1087). Mean ± SEM of *n* = 11–12 mice. ***F***, Density of dentate gyrus BrdU+ cells (cells/μm^2^ × 10^4^) co-labeled with DCX and/or NeuN (two-way repeated measures ANOVA; cell type × TAM: *F*_(2,42)_ = 8.654, *p *=* *0.0007; cell type: *F*_(1.207,25.35)_ = 83.41, *p *<* *0.0001; TAM: *F*_(1,21)_ = 8.675, *p *=* *0.0077; subject: *F*_(21,42)_ = 1.516, *p *=* *0.1239). Mean ± SEM of *n* = 11–12 mice. ***G***, Percent of BrdU+ dentate gyrus cells labeled for DCX and/or NeuN (two-way repeated measures ANOVA; TAM × cell type: *F*_(2,42)_ = 6.501, *p *=* *0.0035; TAM: *F*_(1,21)_ = 16.13, *p *=* *0.0006; cell type: *F*_(1.545,32.44)_ = 151.5, *p *<* *0.0001; subject: *F*_(21,42)_ = 0.3789, *p *=* *0.9903). Mean ± SEM of *n* = 11–12 mice. ***H***, Representative images of BrdU, DCX, and NeuN labeling. Scale bar: 20 μm. ***I***, Density of EdU+ cells (cells/μm^2^ × 10^4^) in the dentate gyrus (unpaired *t* test, *t*_(21)_ = 1.043, *p = *0.3090). Mean ± SEM of *n* = 11–12 mice. ***J***, Density (cells/μm^2^ × 10^4^) of dentate gyrus EdU+ phenotypic RGL-NSCs, IPCs, and other cells (two-way repeated measures ANOVA; cell type × TAM: *F*_(2,42)_ = 1.157, *p *=* *0.3241; cell type: *F*_(1.066,22.38)_ = 210.3, *p *<* *0.0001; TAM: *F*_(1,21)_ = 1.087, *p *=* *0.3090; subject: *F*_(21,42)_ = 0.9645, *p *=* *0.5209). Mean ± SEM of *n* = 11–12 mice. ***K***, Density (cells/μm^2^ × 10^4^) of RGL-NSCs and IPCs in the dentate gyrus (two-way repeated measures ANOVA; cell type × TAM: *F*_(1,21)_ = 1.042, *p *=* *0.3191; cell type: *F*_(1,21)_ = 3.794, *p *=* *0.0649; TAM: *F*_(1,21)_ = 2.404, *p *=* *0.1360; subject: *F*_(21,21)_ = 1.138, *p *=* *0.3846). Mean ± SEM of *n* = 11–12 mice. ***L***, Representative images of EdU, SOX2, and GFAP labeling. Scale bar: 20 μm. *p <* *0.05, **p <* *0.01, ***p <* *0.001, ****p <* *0.0001, as determined by Sidak’s multiple comparisons test.

**Figure 7.**
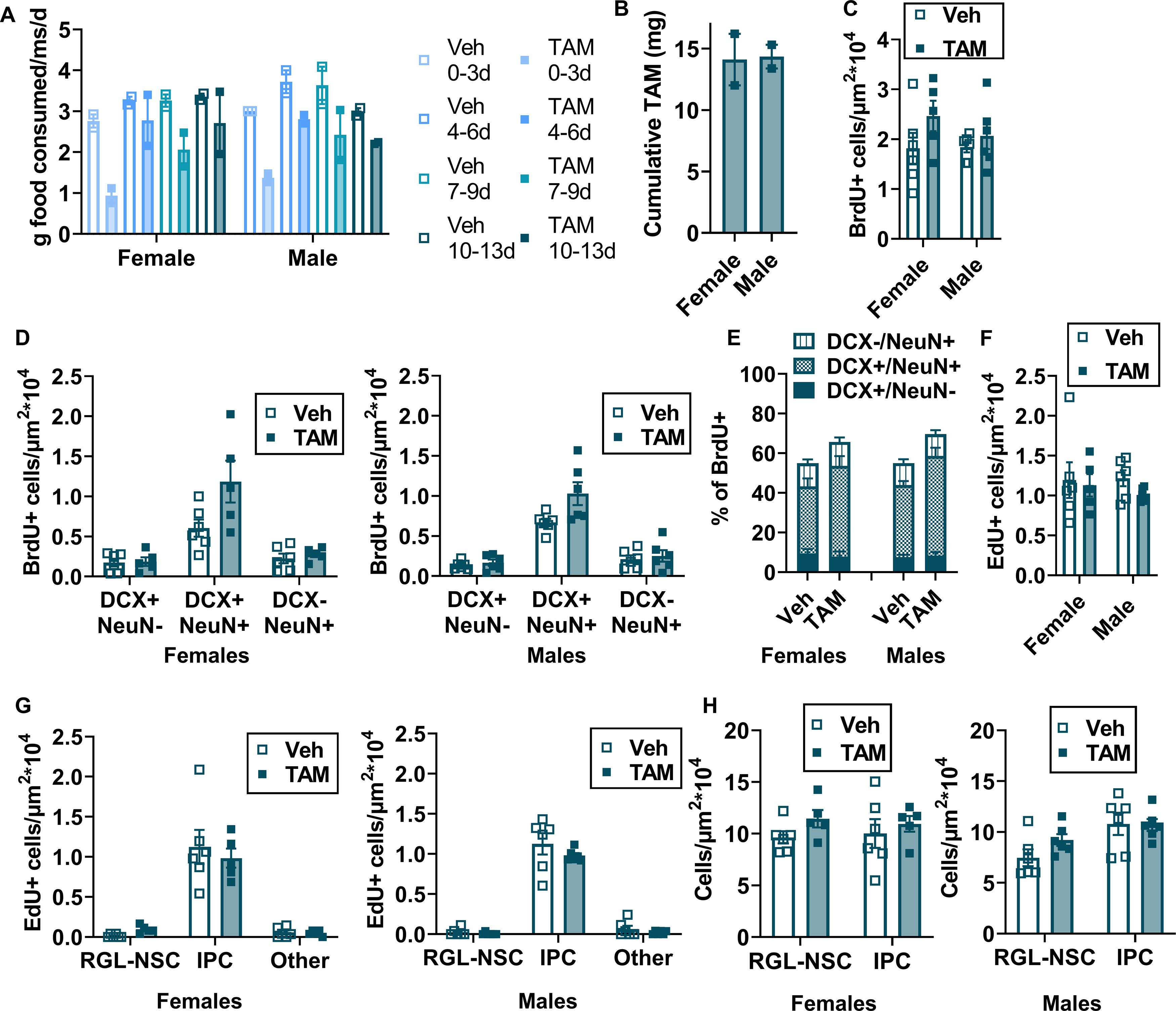
TAM diet effects on cell survival and proliferation do not differ by sex. ***A***, Grams of TAM and vehicle chow consumed by male (*n* = 2 cages/sex) and female (*n* = 2 cages/sex) mice per mouse (ms) per day over each 3- to 4-d period of diet exposure. ***B***, Cumulative TAM consumed in milligrams (mg) by sex (*n* = 2 cages/sex). ***C***, Density of BrdU+ cells (cells/μm^2^ × 10^4^) in the dentate gyrus, by sex (two-way ANOVA; interaction: *F*_(1,19)_ = 0.6818, *p *=* *0.4192; sex: *F*_(1,19)_ = 0.5413, *p *=* *0.4709; TAM: *F*_(1,19)_ = 2.921, *p *=* *0.1037). Mean ± SEM of *n* = 5–6 mice. ***D***, Density of dentate gyrus BrdU+ cells (cells/μm^2^ × 10^4^) co-labeled with DCX and/or NeuN in females and males (three-way repeated measures ANOVA: cell type: *F*_(1.213,23.05)_ = 78.09, *p *<* *0.0001; sex: *F*_(1,19)_ = 0.2586, *p *=* *0.6169; TAM: *F*_(1,19)_ = 8.323, *p *=* *0.0095; cell type × sex: *F*_(2,38)_ = 0.02142, *p *=* *0.9788; cell type × TAM: *F*_(2,38)_ = 8.256, *p *=* *0.0011; sex × TAM: *F*_(1,19)_ = 0.3225, *p *=* *0.5767; cell type × sex × TAM: *F*_(2,38)_ = 0.5548, *p *=* *0.5787). Mean ± SEM of *n* = 5–6 female mice and 6 male mice. ***E***, Percent of BrdU+ dentate gyrus cells labeled for DCX and/or NeuN in male and female mice (three-way repeated measures ANOVA: cell type: *F*_(1.557,29.58)_ = 140.5, *p *<* *0.0001; sex: *F*_(1,19)_ = 0.3592, *p *=* *0.5561; TAM: *F*_(1,19)_ = 14.62, *p *=* *0.0011; cell type × sex: *F*_(2,38)_ = 0.6323, *p *=* *0.5369; cell type × TAM: *F*_(2,38)_ = 5.877, *p *=* *0.0060; sex × TAM: *F*_(1,19)_ = 0.3233, *p *=* *0.5763; cell type × sex × TAM: *F*_(2,38)_ = 0.06,331, *p *=* *0.9387). Mean ± SEM of *n* = 5–6 mice. ***F***, Density of EdU+ cells (cells/μm^2^ × 10^4^) in the dentate gyrus in male and female mice (two-way ANOVA; interaction: *F*_(1,19)_ = 0.2581, *p *=* *0.6173; sex: *F*_(1,19)_ = 0.1190, *p *=* *0.7339; TAM: *F*_(1,19)_ = 0.9220, *p *=* *0.3490). Mean ± SEM of *n* = 5–6 mice. ***G***, Density (cells/μm^2^ × 10^4^) of dentate gyrus EdU+ phenotypic RGL-NSCs, IPCs, and other cells in male and female mice (three-way ANOVA: cell type: *F*_(1.058,20.10)_ = 190.3, *p *<* *0.0001; sex: *F*_(1,19)_ = 0.1190, *p *=* *0.7339; TAM: *F*_(1,19)_ = 0.9220, *p *=* *0.3490; cell type × sex: *F*_(2,38)_ = 0.05511, *p *=* *0.9465; cell type × TAM: *F*_(2,38)_ = 1.094, *p *=* *0.3451; sex × TAM: *F*_(1,19)_ = 0.2581, *p *=* *0.6173; cell type × sex × TAM: *F*_(2,38)_ = 0.08267, *p *=* *0.9208). Mean ± SEM of *n* = 5–6 female mice and 6 male mice. ***H***, Density (cells/μm^2^ × 10^4^) of RGL-NSCs and IPCs in the dentate gyrus of male and female mice (three-way ANOVA: cell type: *F*_(1,19)_ = 3.943, *p *=* *0.0617; sex: *F*_(1,19)_ = 2.181, *p *=* *0.1561; TAM: *F*_(1,19)_ = 2.703, *p *=* *0.1166; cell type × sex: *F*_(1,19)_ = 4.370, *p *=* *0.0503; cell type × TAM: *F*_(1,19)_ = 1.338, *p *=* *0.2618; sex × TAM: *F*_(1,19)_ = 0.1703, *p *=* *0.6845; cell type × sex × TAM: *F*_(1,19)_ = 0.1708, *p *=* *0.6840). Mean ± SEM of *n* = 5–6 female mice and 6 male mice. *p <* *0.05, **p <* *0.01, ***p <* *0.001, ****p <* *0.0001, as determined by Sidak’s multiple comparisons test.

To determine whether TAM chow alters adult neurogenesis, we fed adult wild-type C57BL6/J mice TAM or vehicle-matched chow for 14 d, the last three of which were coupled with once per day BrdU injections to label dividing cells (Fig. 6*A*). Mice were perfused 35 d after the beginning of chow treatment (21 d after the last TAM/BrdU injections), 2 h after a single EdU injection to label acutely proliferating cells. Monitoring of food consumption confirmed that during the first ∼3d of chow exposure, mice consumed significantly less TAM chow than vehicle chow (2.88 ± 0.10 g veh chow/ms/d vs 1.15 ± 0.15 g TAM chow/ms/d), but consumption returned closer to vehicle chow levels shortly thereafter (Figs. 6*B*, 7*A*). Total average TAM consumption per mouse was 14.22 ± 0.95 mg, similar to that seen in Figure 1*E* (Figs. 6*B*, 7*B*). As expected from the TAM chow avoidance, mice that received TAM chow weighed less than vehicle chow-fed mice at the end of chow exposure, although this pattern only reached significance in the male mice (female: 16.65 ± 0.26 g veh chow vs 15.52 ± 0.31 g TAM chow; male: 22.37 ± 0.41 g veh chow vs 18.50 ± 0.45 g TAM chow; Fig. 6*C*,*D*). In females, this pattern reversed after a 21-d recovery and by d35, TAM chow-fed mice weighed significantly more than vehicle chow-fed female mice (18.13 ± 0.25 g veh chow vs 20.00 ± 0.78 g TAM chow; Fig. 6*C*). TAM and vehicle-fed male mice, in contrast, no longer differed in body weight after three weeks of recovery (24.07 ± 0.32 g veh chow vs 24.18 ± 0.88 g TAM mice; Fig. 6*D*).

The total density of surviving BrdU+ cells did not significantly differ between TAM and vehicle chow-fed mice (2.25 ± 0.20 × 10^−4^ cells/μm^2^ vs 1.83 ± 0.16 × 10^−4^ cells/μm^2^, respectively; Fig. 6*E*; Extended Data Fig. 6-1). In contrast, the density of BrdU+ cells co-expressing both DCX and NeuN was significantly higher in TAM chow-fed mice than vehicle-fed mice (0.64 ± 0.06 × 10^−4^ cells/μm^2^ veh chow vs 1.10 ± 0.14 × 10^−4^ cells/μm^2^ TAM chow; Fig. 6*F*,*H*). The percent of BrdU+ cells co-expressing both DCX and NeuN was similarly higher in the TAM chow-fed mice than vehicle chow-fed mice (35.15 ± 2.17% veh chow vs 48.09 ± 3.42% TAM chow; Fig. 6*G*). The percentage of BrdU+ cells single positive for DCX or NeuN was small in both groups and did not differ between groups (DCX: 8.46 ± 1.26% veh chow vs 7.915 ± 1.63% TAM chow; NeuN: 11.38 ± 1.31% veh chow vs 11.42 ± 1.57% TAM chow). Similar results were found when males and females were analyzed separately (Fig. 7*C–E*). These results suggest that the TAM chow exposure paradigm drives more neurogenesis primarily via enhanced neuronal differentiation of cells proliferating during TAM exposure.

10.1523/ENEURO.0422-21.2022.f6-1 Figure 6-1 Raw data supporting Figure 6. Download Figure 6-1, XLSX file.

We also quantified cell proliferation of RGL-NSCs and IPCs three weeks after TAM using EdU to label proliferating cells. EdU+ cell density in the DG did not significantly differ between veh and TAM chow-fed mice (1.21 ± 0.12 × 10^−4^ cells/μm^2^ veh vs 1.06 ± 0.07 × 10^−4^ cells/μm^2^ TAM; Fig. 6*I*,*L*). Classification of EdU+ cells as RGL-NSCs, IPCs or neither similarly showed no difference in EdU+ density of these cell subtypes between chow groups (Fig. 6*J*,*L*). Quantification of total RGL-NSCs and IPCs also showed no difference in total RGL-NSC or IPC density (Fig. 6*K*,*L*). Similar results were found when males and females were analyzed separately (Fig. 7*F–H*). These findings suggest that TAM chow feeding does not strongly alter cell proliferation relative to vehicle chow three weeks after chow treatment has ended.

## Discussion

In this work, we found that TAM chow treated mice showed less recombination efficiency but similar recombination specificity as TAM-injected mice in a commonly used NestinCreER^T2^ model that targets recombination in adult NSPCs. Both administration protocols also had inherent, although different, effects on adult neurogenesis. The TAM injection protocol suppressed IPC proliferation three weeks after TAM, whereas the TAM chow protocol did not disrupt later IPC proliferation but did induce greater neuronal differentiation of cells born during TAM. Altogether, our results suggest that both administration routes of TAM are potentially useful but selection of controls requires attention to the inherent effects of TAM delivery.

The difference between TAM injection and TAM chow in recombination efficiency is most likely explained by total TAM exposure. Recombination efficiency in TAM-inducible transgenic systems is partly dose dependent (Hayashi and McMahon, 2002), and mice given TAM chow were exposed to overall less TAM than those given TAM injections. Mice showed strong aversion to the TAM chow, as evidenced by the approximately one-week delay between chow introduction and substantial daily consumption. It is not surprising then to observe substantially less recombination in chow mice than injected mice. The chow was sweetened to increase palatability and delivered on the cage floor in dishes to ease access, so future efforts to overcome this barrier to TAM ingestion may require longer TAM chow treatments. In studies targeting adult neurogenic processes, however, longer chow treatment will result in a more heterogenous population of recombined cells at different stages of differentiation. Temporal precision is often a goal of adult neurogenesis studies, and extending chow treatment times may not always fit with that goal. Another possibility would be to provide TAM via oral gavage. However, oral gavage is notoriously stressful for animals (Brown et al., 2000) and requires expertise on the part of the experimenter to reduce that stress and avoid injury to animals (Arantes-Rodrigues et al., 2012). If high recombination efficiency and selective targeting of cells of similar maturity are desired, our data suggest that TAM injections are still the preferable method.

Despite differences in recombination efficiency between the TAM administration routes tested here, this work also shows that two weeks of TAM chow induces genetic recombination with similar specificity to 5 d of TAM injection. Mice fed TAM chow for two weeks showed a recombined cell population of similar phenotype (primarily NSPCs) as TAM-injected mice 7 d after the start of injections. Thus, TAM-infused chow could be a workable alternative administration route when TAM injection is not feasible, when lower recombination efficiency is desired, or when long-term suppression of cell proliferation would interfere with interpretation of experimental results.

Our findings comparing TAM versus vehicle-injected mice provide several useful insights into the effects of this commonly-used agent on adult neurogenesis. First, we found that neurogenesis derived from cells born during TAM was not substantially affected by TAM injection. These results are similar to those reported in (Rotheneichner et al., 2017), which showed no difference in BrdU+ cell density or phenotype between TAM and vehicle-injected mice 10 d after TAM/BrdU using five-month-old male and female mice. In contrast, using three- to four-week-old (juvenile) male mice, Lee et al. (2020) found that the density of surviving BrdU+ cells approximately one week after TAM/BrdU was suppressed by ∼2-fold in TAM-injected versus vehicle-injected mice. Combined, these findings suggest that the effect of TAM injection on production of new DG cells that survive 1+ weeks could depend on organismal age. Sex may also be a factor, but this is difficult to discern as previous work either used only males or did not show data for males and females separately. Future research is needed to address the effects of TAM on cell proliferation and survival to better understand how TAM affects neurogenesis across the lifespan and in both sexes.

Second, we show that TAM injection had prolonged effects on progenitor cell proliferation, suppressing progenitor density and proliferation by ∼1.3-fold three weeks after TAM. Lee and colleagues similarly reported a ∼1.8-fold TAM-induced suppression of Mki67-labeled progenitors one week after TAM in juvenile mice. Rotheneichner et al. (2017) reported no difference in PCNA+ progenitor cell number 10 d after TAM in their five-month-old mice, however. Again, this difference may reflect increased susceptibility of juvenile mice to the effects of TAM, a subtle sex difference, or a time point effect.

The mechanism by which TAM suppresses progenitor proliferation weeks after TAM treatment is unclear. One possible mechanism for suppression of cell proliferation associated with TAM treatment in general is brain estrogen receptor modulation by TAM. TAM acts on brain ERα, ERβ, and the transmembrane receptor GPR30 (Gonzalez et al., 2016). Specifically, TAM can be an estrogen agonist at GPR30 (Filardo et al., 2000; Vivacqua et al., 2006a,b) and ERβ (McDonnell and Wardell, 2010), and it can induce estrogen blockade at ERα (McDonnell and Wardell, 2010). Because ERα agonists can enhance cell proliferation in the adult hippocampus (Mazzucco et al., 2006), it therefore seems possible that TAM blockade of ERα could suppress proliferation. Receptor-independent effects on cell cycle genes, as proposed by Lee et al. (2020), are also a potential mechanism of TAM-induced suppression of proliferation. With either mechanism, however, it is unclear why those effects would persist long after TAM withdrawal. TAM metabolites remain in the brain for ∼8 d after administration (Valny et al., 2016), making it unlikely that suppression of proliferation three weeks later is a result of active TAM presence. This long-term persistence could therefore be because initial effects of TAM are slow to arise and/or indelible (e.g., epigenetic changes), or because they reflect effects of TAM withdrawal rather than TAM itself. Future research is needed to parse out these multiple, nonexclusive hypotheses.

We also noted delayed weight gain in female mice that received TAM injection, resulting in higher average body mass of TAM treated females than vehicle treated females three weeks after injection. Because TAM was administered systemically, this delayed weight gain in female mice could be because of TAM effects on multiple central or peripheral tissues. One candidate tissue is the hypothalamus. In mice, hypothalamic ERα-dependent gene expression changes have been linked to common TAM side effects, including decreased movement (Zhang et al., 2021), which could contribute to weight gain.

TAM chow had qualitatively different effects on neurogenesis and cell proliferation than TAM injection. In contrast to TAM injections, TAM chow increased neuronal differentiation of cells born during TAM but had no effect on density of EdU+ cells three weeks after TAM. Caloric restriction and reduced total TAM exposure in chow-fed mice (compared with TAM-injected mice) both likely contribute to this difference between paradigms. Caloric restriction is a particularly important confounding factor to consider when comparing TAM chow-fed to vehicle chow-fed animals. Although Bondolfi and colleagues found no effect of caloric restriction on hippocampal neurogenesis in male mice aged 3–11 months (Bondolfi et al., 2004), others have found that dietary restriction and intermittent fasting enhance neuronal differentiation in the adult hippocampus (Hornsby et al., 2016; Li et al., 2020). Both the caloric restriction during TAM feeding and the return to normal feeding after the end of TAM chow may therefore be affecting neurogenesis in TAM-fed mice. Regardless of the reason for differing effects of TAM by administration route, our data further underscore the need for internal TAM-treated controls in experiments using TAM chow, just as with TAM injection.

### Limitations and future directions

Because mice often crumble food and spread it across the floor of the cage, we are unable to account for chow that was chewed in this manner but not consumed. Our calculations of the amount of TAM chow consumed are almost certainly overestimations. This is a limitation of using chow that others may want to consider when knowing exact TAM amounts is critical to study design or interpretation.

Because this work examines direct TAM effects on experimental endpoints only in the context of neurogenesis research in adult animals, further testing is needed to determine the suitability of alternate TAM dosing methods for subadult animals and research on other brain processes. Also, our BrdU+ counts reflect both cell proliferation during TAM administration and post-TAM cell survival, making it difficult to disentangle TAM effects on either process independently. Thymidine analogs, such as the BrdU and EdU we used here, mark only a subset of proliferating cells, so it is possible that further or fewer differences would be observed if all proliferating cells were labeled (with a mitotic marker such as PCNA or MCM2) and assessed. It is also possible that any or all of the effects observed in this study could vary throughout the anatomy of the dentate gyrus (dorsal vs ventral, inferior vs superior blades of the subgranular zone). Our counts reflect areas sampled throughout the DG and therefore do not capture this information. In addition, although we provide the parental source of NestinCreER^T2^ in Extended Data Figure 2-1, we do not have sufficient sample size to draw conclusions about the effect of inheriting maternal versus paternal NestinCreER^T2^ on any of the outcome measures.

Better understanding of the molecular mechanism by which TAM acts on adult neurogenesis, when provided by injection or chow, is also still needed, as is further assessment of how (if at all) TAM differentially affects reporter-labeled and nonlabeled cell populations. Finally, clarity regarding the actions of TAM on brain estrogen receptors is lacking and could help guide researchers’ choice of TAM dosing methods and interpretation of studies where TAM is used.

In conclusion, this work shows that voluntary TAM chow consumption may be a suitable alternative to TAM injections for inducing Cre-lox recombination in some adult neurogenesis studies. This work further identifies several effects of TAM administration protocols, whether by injection or food, on adult neurogenesis endpoints. These effects are separate from genetic recombination effects and are an important confounding variable in experimental designs that rely on comparisons to a vehicle-treated control. Thus, we suggest that research using TAM-inducible Cre lines use TAM-treated wild-type (nonrecombination susceptible) littermates as controls, rather than vehicle-treated mice.

## Synthesis

Reviewing Editor: Deanna Smith, University of South Carolina Decisions are customarily a result of the Reviewing Editor and the peer reviewers coming together and discussing their recommendations until a consensus is reached. When revisions are invited, a fact-based synthesis statement explaining their decision and outlining what is needed to prepare a revision will be listed below. The following reviewer(s) agreed to reveal their identity: Mirjana Maletic-Savatic. Note: If this manuscript was transferred from JNeurosci and a decision was made to accept the manuscript without peer review, a brief statement to this effect will instead be what is listed below.

Both reviewers agreed that the revised version is much improved. This paper addresses questions that are either overlooked (inappropriately) by the field or are asked on an individual lab basis. The field will welcome this information on such a fundamental “reagent” in neuroscience and neurogenesis research. The data also give traction to those who attempt to address discrepancies in the literature - route of Tam is particularly important to consider.The advance is not major, but puts different modes of tamoxifen use in perspective. Tamoxifen is widely used to induce Cre in conditional transgenic mice and as such, the knowledge provided by this work should be useful for a wide community.

## Author Response

Reviewer 1

This manuscript provides timely and relevant information: guidance for selection of TAM administration route and appropriate controls in adult neurogenesis studies using TAM-inducible Cre mice. The writing and data presentation are excellent; very logical flow in introduction, thoughtful naming of groups and selection of colors, clear schematics, outstanding images, sharp presentation of data (showing individual mice or cages). The abstract in particular is exceptional in its summary of the many faceted data and pointing out key take home messages for the field. The conclusions, though, are difficult to currently support given that many things are absent from (perhaps due to space consideration) the manuscript, such as information relevant to breeding, chow composition (this is particularly important), data collection and analysis. There are points in the Results and Discussion that merit revisiting, such as the sole reliance on density for outcome measures, clarification of where in the hippocampus these values were collected, and precision of word choice. Comments are listed below by subsection and as major/minor.

Introduction, minor:

As the manuscript does not address optimal dosing schedule (only one dosing schedule is used), perhaps this phrase can be removed.

Author response: We removed this phrase and replaced it with, “appropriate dosing schedules.”

Reviewer 1

Methods, major:

Provide breeding, parental, offspring, and genotype details. Which parent provided the cre? Were the offspring only carrying one copy of each parent gene? Was there a relationship with any of these factors, sex, and the output measures in this work? Were the offspring 8-9 weeks old (as said in Methods) or 7-9 weeks old (as in schematic)? Each of these points influences the data and their interpretation.

Author response: In study 1, we crossed C57BL/6J wildtype mice with mice homozygous for both NestinCreERT2 and R26R-LoxP-STOP-LoxP-EYFP to produce subject mice who were heterozygous for both NestinCreERT2 and R26R-LoxP-STOP-LoxP-EYFP. We have added these details in the methods section. Our studies had a mix of animals with maternally and paternally inherited Cre. However, subdividing our data even further along these lines leads to such small sample sizes that the data are not readily interpreted. For example, only one subject in the day 14 injection group received maternal Cre. We have, however, provided a new table (Table 3) that has this information along with raw data for each mouse in study 1 for others to use and draw conclusions from as they wish. We have also added a note about this limitation in the Limitations section. The offspring were 7-9 weeks old in study 1. They were 8 weeks old at the study start for studies 2 and 3. This is indicated now in the schematics and methods.

Reviewer 1

Provide a full nutritional profile of “regular” chow, vehicle, and TAM chow. Does consideration of these profiles influence how the authors interpret their data? Since the injectable form of TAM was protected from light and refreshed after 1 week, what (if any) precautions were taken with the TAM chow? Are there additional limitations that emerge from the answers to these questions?

Author response: We have added these details in the methods. Standard chow, Vehicle chow and TAM chow all contain 15.4% protein, 55.1% carbohydrate, and 3.4% fat by weight. Vehicle and TAM chow also contain 50 g/Kg of sucrose and 0.25 g/Kg of blue or red food color; TAM chow also contains 0.5 g/Kg of TAM. TAM chow contains extra sugar (compared with standard chow) to boost palatability; vehicle chow is formulated the same way as sweetened chow is a more appropriate control diet than standard, less sweet chow. To preserve the TAM chow, we stored it at 4℃ in the dark and refreshed it every 3 days (as recommended by the manufacturer).

Reviewer 1

It is stated that TAM chow consumption is reported by cage/number of animals in the cage. Given that chow crumbles, mice often chew without consuming, and the TAM chow is not weighed every day, is it possible that a meaningful amount of chow was not actually “consumed” but rather was just chewed off and left as crumbs in the bedding? Might this lead to a different reported measure (maybe the consumed amount is an overestimation?) or conclusion, or an additional limitation? (a related minor note: do studies that use special chow typically just switch to the special chow, as was done here, or is a habituation period with the vehicle chow used?)

Author response: With this study, we aimed to assess the feasibility of TAM administration through chow. It’s likely that a meaningful amount of chow was not consumed. We have clarified this limitation in the Limitations section, in the Results and we have renamed the Y axis of Fig 1D to be “g food eliminated/ms/d”. Some groups fast mice following the removal of standard chow and the introduction of TAM-infused chow to encourage mice to consume the TAM chow (see for example Yoshinobu et al., Experimental Animals, 2021). Other articles do not clarify whether habituation or fasting are used to encourage the consumption of TAM diet (e.g. Kiermayer et al.; Wang et al., JNeuro, 2017; Song et al., PNAS, 2020). Ceasrine et al. (Endocrinology, 2019) appear to have switched mice directly from standard chow to either control or TAM-infused chow, with a 2-day period on standard chow halfway through their 10-day TAM diet protocol. We acclimated mice to delivery of standard chow on the cage floor in a dish for 3 days before switching to experimental diet. This is described in the methods.

Reviewer 1

The hippocampus is a large structure, even in the mouse. Many studies have shown different levels of new neurons or other marks of neurogenesis in different aspects of the dentate gyrus (the septal vs. temporal, medial vs. lateral, and even the upper granule cell layer vs. the lower one; some papers even suggest left vs. right differences). The data here are just presented as density but it is not clear where in the dentate gyrus these data come from. Which aspect(s) of the hippocampus was/were the focus/foci of the present study? If all of the hippocampus was studied and averaged together, what other conclusions are evident once the data in these different/separate aspects of the hippocampus are considered? For example, is the lower density of recombined cells in chow vs. injected mice more “significant” in one part of the dentate gyrus and less in another? Or is this a uniform decrease?

Author response: We did not subdivide the DG along these dimensions. We have added that as a limitation of the study.

Reviewer 1

The route of TAM (injection vs. chow) is not used as a variable in the statistical model (eg, Fig 2B). It is unclear why the data are compared via one-way ANOVA when there are statistical approaches to test if route of TAM (injection vs. chow) is indeed a factor contributing to differences (even though the endpoints are distinct: d7, d14 for injection, and 10d, 14d for chow). Sex is sometimes considered a variable in the statistical model (eg, food consumption, sex x day) and other times it is not (eg, weight, mixed model of tam route x day performed separately in the sexes without first testing if there is a sex effect to rationalize showing them separately). While there are excellent female vs. male analyses performed in the manuscript, the lack of consistency in how sex is considered is confusing. Related to statistics: Subject number varies across graphs, but it is not clear why; perhaps space limitations preclude full explanation. The name of the statistical software is not provided.

Author response: In Study 1, we debated use of one-way ANOVA vs two-way ANOVA with factors being delivery method x timepoint. This approach would lead to equating the time points between chow and injection. For 14d, this equivalency is could be argued for based on the number of days since TAM start being the same for injection and chow (though we did not actually mean to imply equivalency of the two time points as the delay in TAM chow consumption makes days since start not the most readily comparable measure). But the earlier time points are not equivalent even in number of days (7d injection and 10d chow). We decided we were not comfortable with making that equivalency apriori-when we began these studies, we did not know which timepoints should actually be equivalent and that was one of our more open experimental questions. We also originally had hoped to have 7d be our first chow time point but after observing how little chow was eliminated from the cages by day 7, we chose to extend the chow several more days. We now provide raw data in Table 3 which could allow readers to perform a 2 way ANOVA should they wish to do so. Statistical analyses were performed in GraphPad Prism (version 9.3.1).

We did not make sex a variable in measures of body weight because we expected strong main effects of sex there which would not be of particular interest but could mask experimental effects if part of an overall ANOVA. A note about this has been added to the methods. In the figures dedicated to separating brain data by sex, sex was always a variable, including when used as part of 3-way ANOVAs in Fig 5E,F and 7D,E,G,H.

For the varying number of subjects, we are not sure where the reviewer is seeing this. Some measures are reported as whole cage, resulting in n = cage, not n = mice. This is now added to the methods section under Cell quantification subsection. The new Table 3 also has a great deal of raw data in it and now data are missing across subjects. Could the reviewer be more specific on where they see missing data?

Reviewer 1

Results, Major:

Throughout density is used to report outcome measures, which is beneficial in that it normalizes for area sampled. However, this assumes that the areas sampled do not change. Can the authors also provide the areas sampled and number of the cells counted rather than only density? This would not only address a potential concern about area being influenced by Tam injection or chow (unlikely, but possible) but would make it more feasible to compare the values presented here with those more broadly published in the literature by this and other groups.

Author response: This data is now provided in the new Table 3.

Reviewer 1

Another important density note: as density is reported, the conclusions should use the word density, not number. For example, “these results suggest that TAM injection during BrdU-labeling does not alter the number of new-born neurons 3 weeks later” is not accurate, but “these results suggest that TAM injection during BrdU-labeling does not alter the density of new-born neurons 3 weeks later” is. Also, “These results suggest that the TAM chow exposure paradigm drives more neurogenesis primarily via enhanced neuronal differentiation of cells proliferating during TAM exposure” is not accurate, but “These results suggest that the TAM chow exposure paradigm increases the density of....” is. This distinction between density and number should be made throughout the manuscript.

Author response: We have corrected the phrasing in these lines to be more accurate.

Reviewer 1

There are several phrases where the wording is not appropriate given the statistics and data. Just one example: “Both male and female TAM chow-fed mice showed significant weight loss compared to TAM-injected mice”: Based on the data and the statistics reported, the TAM chow-fed mice weigh less than TAM-injected mice, but do not have “weight loss” vs. Tam-injected mice. In fact, it looks more like the TAM chow-fed mice do not gain weight overtime, but this is not assessed in the posthoc testing shown (eg, later timepoints are not compared to earlier timepoints within group). This is evident again in Figure 1 title (“reduces body mass”) and in other similar statements in the Results (although in regards to body weight, the data are reviewed in the Discussion using the appropriate terms). Check throughout for such discrepancies.

Author response: We have corrected these lines in the results, as well as the title of figure 1 to clarify that TAM chow seems to have prevented weight gain rather than causing weight loss.

Reviewer 1

Clarify n throughout. For example, in Fig 1B and C the n is 7-11 but Fig 1E the n is 11-13 (injection). Why aren’t the n the same throughout? What happened to the mice omitted? Which groups are they from? Are the same 7-11 mice used throughout all the other figures?

Author response: There is an error here and the legend for Fig 1B/C should actually have a bigger n variance: n = 4-13. This apparent difference with what is shown in Fig 1E is because of how the weight data has data points with as few as n = 4 (on day 14, when animals from day 7 injection and day 10 chow are no longer in the study) and as many as 13 (when all animals are in the study). A line has been added to clarify this in the figure legend (and the n has been corrected). The new raw data in Table 3 also provides much more clarity about n.

Reviewer 1

"Adult wildtype C57BL6/J mice were injected with TAM or matched volumes of vehicle (oil)"; should this be “match dose (ml/kg) of vehicle", not volume?

Author response: We have altered the phrasing to read, “Adult wildtype C57BL6/J mice were injected with TAM or matched dose (ml/kg) of vehicle (oil) for 5d.”

Reviewer 1

In regard to an issue raised by Reviewer 2 (below) I question whether the estrous cycle would need to be tracked. Many researchers have shown neurogenesis measures don’t change throughout the female C57Bl/6J estrous cycle (although others say it does, and it definitely appears to change in the rat). A useful review on this is Rebecca Shansky’s “Are hormones a “female problem” for animal research?” (Science, 31 MAY 2019, VOL 364 ISSUE 6443) which points out that there may be as much (or more) variation in males than in females. Also, I don’t think the data in this manuscript suggest a lot of within-sex variation.

Author response: We thank reviewer 1 for these comments and have further addressed this issue beneath reviewer 2’s relevant comment.

Reviewer 1

Results, Minor:

When comparing males and females, use of the same scale of Y axis makes comparison more straightforward.

Author response: We found one y-axis where this needed correction (Fig 7G). If there are others we missed, please let us know.

Reviewer 1

Rather than “mice were randomly assigned", use the phrase from the methods: “cages of mice were randomly assigned”.

Author response: We have made this change in the methods.

Reviewer 1

While the authors quantified cell proliferation of (or rather density of proliferating) RGL-NSCs and IPCs 3 weeks after TAM using EdU to label proliferating cells, this is an (understandably) narrow outcome measure in that it only assesses EdU labeled cells. In this sense, these data aren’t reflective of “proliferation” of all the cells (eg, Ki67 or PCNA cells) but rather only the EdU labeled population. It is very possible that proliferation of all the cells (Ki67, PCNA cells) will be differentially influenced than the more limited population of EdU cells. Taking this to a slightly different level: More globally, knowing how Tam influences both the “targeted"/EYFPlabeled population vs. the non-EYPlabeled population would add a lot to our understanding of what researchers are actually influencing when tam is administered.

Author response: This issue is an interesting one, and we have clarified that EdU labels only a subset of proliferating cells in the limitations section. Exploring any differences between reporter-labeled and non-labeled populations following TAM is now listed as a future direction in discussion.

Reviewer 1

Discussion, minor:

Was the assumption that d14 after the start of Tam injections or 14d of Tam chow would be equivalent in regard to the recombination? Given that less Tam reaches the blood and thus brain via chow vs. ip, the conclusion - “lower, but detectable, LoxP- recombination following 10-14d of TAM chow feeding compared to a standard 5d injection regimen” - does not seem surprising, particularly since they essentially didn’t eat much early in the administration period. This point might be made more strongly in the Discussion.

Author response: When we started these studies, we genuinely hoped TAM chow would be a viable alternative to injections. We knew of no data about using TAM chow in these models. When we first observed how little food was consumed, we hoped extending to 2 weeks might deliver enough TAM to get substantial recombination. This turned out to not be the case-TAM chow, even for 2 weeks, does not produce recombination on the same scale as injections. We have added a line about the lower recombination not being surprising given the low consumption to the discussion.

Reviewer 1

Building off of the last major Results comments, the Tam used in these studies influences cells that express ERT2 but likely has direct and indirect effects on other cells as well (as the authors know, since they have a nice ER discussion paragraph). The authors may want to include a brief summary of tam effects seen in non-ERT2 expressing cells and animals (which is a bit broader than the summary provided).

Author response: We have added this to the discussion.

Reviewer 1

As Tam is lipophilic, might it remain in fat stores and release over time? The brain is of course very fatty, and Tam only appears to stay there 8 days, but perhaps the cancer field has some insight about tam’s long-lasting effects (and whether it might store in other fat sources in the body).

Author response: A study by Jahn et al. shows TAM peaks in brain ∼8h after injection and is cleared to ineffective levels by 7d (Jahn et al., (2018) 8:5913 | DOI:10.1038/s41598-018-24085-9). Repeated injections of TAM show a longer half-life in brain but are still almost absent by 7d after last injection.

OH-tamoxifen is used in many systems to provide a more robust recombination. Is it known if OH-Tam works “better” in the resulting mouse used here?

Author response: To our knowledge, it isn’t known whether OH-Tam works better in the mouse line we used here.

Reviewer 1

Different reporter lines lead to different outcomes. Is it known if the cre driver used here gives either a different pattern or higher efficiency of recombination in another reporter mouse?

Author response: A previous study out of our lab addressed this question (Dause & Kirby, eNeuro, 2020). Using a NestinCreERT2;Rosa(EYFP/tdTom) mouse, we found that, both recombination efficiencies of the two reporters were comparable as were the phenotype of cells the evidenced recombination in each reporter line. Interestingly, though both reporters were on average detected in the same types of cells (RGL-NSCs and IPCs), the concordance within individual cells was low, suggesting a stochastic process in which each transgene has a probability <100% of undergoing recombination within a cell that is expressing NestinCreERT2 and is exposed to TAM.

Reviewer 2

This is a straightforward paper that addresses whether tamoxifen affects neurogenesis and whether there is any difference if it is given IP or orally. In essence, the answers to the above questions are provided in simple experiments and conclusive results. However, there are several other papers published on the same issue and the rationale and novelty of this particular study is not clear. Furthermore, tamoxifen in food cannot be traced to ensure that the experimenter knows exactly how much is ingested; this leads to high variability of outcome measures. In turn, oral gavage - which will give at least consistent dose - is uncomfortable for animals compared to IP. So, I am not sure about the practical value of this study and whether it brings new knowledge that would affect the field or change practice in the field.

Author response: Reviewer 2 makes strong points about the possible drawbacks of TAM chow as an administration method. All drug administration methods have downsides to consider, and our goal in conducting these experiments was to determine whether TAM chow is a workable method for adult neurogenesis research despite the method’s inherent imprecision. We present the data so future investigators won’t each have to do this for themselves. Rather, they can use our data to decide if TAM chow will work for them. As noted in response to reviewer 1, we started this hoping that chow would work well. We were disappointed and hope to save others the trouble if the barriers we documented mean chow will not help them achieve their experimental goals. There is now more discussion of this purpose in the discussion section.

Reviewer 2

Furthermore, authors perform analysis of male and female mice. Given that TMX modulates estrogen receptor, it is necessary to note when the female mice were treated with it with respect to their menstrual cycle. As authors discuss in this paper, adult hippocampal neurogenesis is affected by a myriad of different (physiological) factors; in addition, it is known that the number and plasticity of neuronal spines oscillate in female mice depending on the estrogen levels, so administration of TMX should be consistent to avoid these confounders.

Author response: Increased variability of female rodents relative to male rodents is an interesting question that has been addressed by several recent articles, including (as reviewer 1 mentioned) “Are hormones a “female problem” for animal research?” (Science, 31 MAY 2019, VOL 364 ISSUE 6443). Jill Becker’s 2016 meta-analysis “Female rats are not more variable than male rats: a meta-analysis of neuroscience studies” also concluded that female rats are no more variable than male rats across a variety of outcome measures. Furthermore, estrous cycles in mice last 4-5 days, thus female mice in both the injection and chow groups were treated with tamoxifen across all stages of the cycle.

Reviewer 2

Specific comments:

Tamoxifen is not a synthetic estrogen but a selective estrogen receptor modulator (SERMs). SERM are a class of drugs that function like estrogen in some tissues but block estrogen’s actions in others. Please correct.

Author response: We have changed the phrasing in the abstract and introduction to correct this issue.

Reviewer 2

How many mice were in the same cage that was given TMX in chow? Unless they were single-caged, there is no way to know how much TMX they injected; but single-caged isolation would affect neurogenesis on its own. Thus, the calculation of the fold change in ingestion could be misleading as some mice may actually have much higher (or lower) levels in blood.

Author response: Each cage contained 3-5 mice. Possible within-cage variability is a potential drawback of TAM chow, and our motivation for conducting these experiments was to find out whether the method works despite its limitations. The imprecision of TAM consumption is addressed with more emphasis in the results and discussion now.

Reviewer 2

Fig 1 E shows the cumulative dose of Tamoxifen on day 7 and day 14. How was this calculated?

Author response: The cumulative dose of tamoxifen for injections was calculated by adding the number of milligrams in each injected dose for each mouse over the 5 days of the injection protocol. This point has been clarified in the methods.

Reviewer 2

In Fig 2H, eYFP+ cells are represented by green, DCX+ cells by Magenta but what is the marker of white-colored cells?

Author response: White is the result of green and magenta mixing so this is label overlap. This is follows a color-pallet that we understand to be preferred at eNeuro as it is more accessible to color-blind readers.

Reviewer 2

In a similar paper ‘Tamoxifen Activation of Cre-Recombinase Has No Persisting Effects on Adult Neurogenesis or Learning and Anxiety’ by Rotheneichner et al. (doi.org/10.3389/fnins.2017.00027), authors have shown that oral gavaging has similar efficiency of recombination as i.p. and is also more beneficial than i.p. as it reduces any chance of inflammation caused by corn oil accumulation in the peritoneal cavity. How do authors correlate their study with the published report as in the current study authors show that oral administration leads to low recombination.

Author response: With oral gavage, as with i.p. injections, investigators can precisely control the dose of TAM administered. Administration of TAM through chow, however, somewhat depends on voluntary consumption by the mice. Our data show that mice avoid consuming the TAM chow for the first 5 days that it is offered. Therefore, lower recombination following TAM chow relative to TAM injection is probably due to lower total TAM exposure, whereas TAM exposure is likely roughly equivalent between gavage and injection groups in Rotheneichner et al. This avoidance of TAM and lower recombination with chow is one of our primary outcome measures. We hope our reporting of these results will save other investigators from having to replicate this work themselves and rather be able to chose a TAM dosing strategy that fits their needs.

## References

[B1] Arantes-Rodrigues R, Henriques A, Pinto-Leite R, Faustino-Rocha A, Pinho-Oliveira J, Teixeira-Guedes C, Seixas F, Gama A, Colaço B, Colaço A, Oliveira PA (2012) The effects of repeated oral gavage on the health of male CD-1 mice. Lab Anim (NY) 41:129–134. 10.1038/laban0512-129 22517091

[B2] Bondolfi L, Ermini F, Long JM, Ingram DK, Jucker M (2004) Impact of age and caloric restriction on neurogenesis in the dentate gyrus of C57BL/6 mice. Neurobiol Aging 25:333–340. 10.1016/S0197-4580(03)00083-6 15123339

[B3] Brown AP, Dinger N, Levine BS (2000) Stress produced by gavage administration in the rat. Contemp Top Lab Anim Sci 39:17–21. 11178310

[B4] Danielian PS, Muccino D, Rowitch DH, Michael SK, McMahon AP (1998) Modification of gene activity in mouse embryos in utero by a tamoxifen-inducible form of Cre recombinase. Curr Biol 8:1323–1326. 10.1016/S0960-9822(07)00562-3 9843687

[B5] Dause TJ, Kirby ED (2020) Poor Concordance of floxed sequence recombination in single neural stem cells: implications for cell autonomous studies. eNeuro 7:ENEURO.0470-19.2020. 10.1523/ENEURO.0470-19.2020 PMC7086402 32079584

[B6] Feil R, Wagner J, Metzger D, Chambon P (1997) Regulation of Cre recombinase activity by mutated estrogen receptor ligand-binding domains. Biochem Biophys Res Commun 237:752–757. 10.1006/bbrc.1997.7124 9299439

[B7] Filardo EJ, Quinn JA, Bland KI, Frackelton AR Jr (2000) Estrogen-induced activation of Erk-1 and Erk-2 requires the G protein-coupled receptor homolog, GPR30, and occurs via trans-activation of the epidermal growth factor receptor through release of HB-EGF. Mol Endocrinol 14:1649–1660. 10.1210/mend.14.10.0532 11043579

[B8] Gonzalez GA, Hofer MP, Syed YA, Amaral AI, Rundle J, Rahman S, Zhao C, Kotter MRN (2016) Tamoxifen accelerates the repair of demyelinated lesions in the central nervous system. Sci Rep 6:31599. 10.1038/srep31599 27554391 PMC4995517

[B9] Hayashi S, McMahon AP (2002) Efficient recombination in diverse tissues by a tamoxifen-inducible form of Cre: a tool for temporally regulated gene activation/inactivation in the mouse. Dev Biol 244:305–318. 10.1006/dbio.2002.0597 11944939

[B10] Hornsby AKE, Redhead YT, Rees DJ, Ratcliff MSG, Reichenbach A, Wells T, Francis L, Amstalden K, Andrews ZB, Davies JS (2016) Short-term calorie restriction enhances adult hippocampal neurogenesis and remote fear memory in a Ghsr-dependent manner. Psychoneuroendocrinology 63:198–207. 10.1016/j.psyneuen.2015.09.023 26460782 PMC4686051

[B11] Jorgensen C, Wang Z (2020) Hormonal regulation of mammalian adult neurogenesis: a multifaceted mechanism. Biomolecules 10:1151. 10.3390/biom10081151 PMC7465680 32781670

[B12] Kiermayer C, Conrad M, Schneider M, Schmidt J, Brielmeier M (2007) Optimization of spatiotemporal gene inactivation in mouse heart by oral application of tamoxifen citrate. Genesis 45:11–16. 10.1002/dvg.20244 17216603

[B13] Lagace DC, Whitman MC, Noonan MA, Ables JL, DeCarolis NA, Arguello AA, Donovan MH, Fischer SJ, Farnbauch LA, Beech RD, DiLeone RJ, Greer CA, Mandyam CD, Eisch AJ (2007) Dynamic contribution of Nestin-expressing stem cells to adult neurogenesis. J Neurosci 27:12623–12629. 10.1523/JNEUROSCI.3812-07.2007 18003841 PMC3718551

[B14] Lee CM, Zhou L, Liu J, Shi J, Geng Y, Liu M, Wang J, Su X, Barad N, Wang J, Sun YE, Lin Q (2020) Single-cell RNA-seq analysis revealed long-lasting adverse effects of tamoxifen on neurogenesis in prenatal and adult brains. Proc Natl Acad Sci U S A 117:19578–19589. 10.1073/pnas.1918883117 32727894 PMC7431037

[B15] Li W, Wu M, Zhang Y, Wei X, Zang J, Liu Y, Wang Y, Gong C-X, Wei W (2020) Intermittent fasting promotes adult hippocampal neuronal differentiation by activating GSK-3β in 3xTg-AD mice. J Neurochem 155:697–713. 10.1111/jnc.15105 32578216

[B16] Mazzucco CA, Lieblich SE, Bingham BI, Williamson MA, Viau V, Galea LAM (2006) Both estrogen receptor α and estrogen receptor β agonists enhance cell proliferation in the dentate gyrus of adult female rats. Neuroscience 141:1793–1800. 10.1016/j.neuroscience.2006.05.032 16797852

[B17] McAvoy KM, Sahay A (2017) Targeting adult neurogenesis to optimize hippocampal circuits in aging. Neurotherapeutics 14:630–645. 10.1007/s13311-017-0539-6 28536851 PMC5509633

[B18] McDonnell DP, Wardell SE (2010) The molecular mechanisms underlying the pharmacological actions of ER modulators: implications for new drug discovery in breast cancer. Curr Opin Pharmacol 10:620–628. 10.1016/j.coph.2010.09.007 20926342 PMC2981619

[B19] Miller SM, Sahay A (2019) Functions of adult-born neurons in hippocampal memory interference and indexing. Nat Neurosci 22:1565–1575. 10.1038/s41593-019-0484-2 31477897 PMC7397477

[B20] Rotheneichner P, Romanelli P, Bieler L, Pagitsch S, Zaunmair P, Kreutzer C, König R, Marschallinger J, Aigner L, Couillard-Després S (2017) Tamoxifen activation of Cre-recombinase has no persisting effects on adult neurogenesis or learning and anxiety. Front Neurosci 11:27. 10.3389/fnins.2017.00027 28203140 PMC5285339

[B21] Semerci F, Maletic-Savatic M (2016) Transgenic mouse models for studying adult neurogenesis. Front Biol (Beijing) 11:151–167. 10.1007/s11515-016-1405-3 28473846 PMC5412727

[B22] Srinivas S, Watanabe T, Lin C-S, Tanabe Y, Jessell TM, Costantini F (2001) Cre reporter strains produced by targeted insertion of EYFP and ECFP into the ROSA26 locus. BMC Dev Biol 1:4. 11299042 10.1186/1471-213X-1-4 PMC31338

[B23] Sun MY, Yetman MJ, Lee TC, Chen Y, Jankowsky JL (2014) Specificity and efficiency of reporter expression in adult neural progenitors vary substantially among nestin-CreER(T2) lines. J Comp Neurol 522:1191–1208. 10.1002/cne.23497 24519019 PMC4047796

[B24] Toda T, Parylak SL, Linker SB, Gage FH (2019) The role of adult hippocampal neurogenesis in brain health and disease. Mol Psychiatry 24:67–87. 10.1038/s41380-018-0036-2 29679070 PMC6195869

[B25] Valny M, Honsa P, Kirdajova D, Kamenik Z, Anderova M (2016) Tamoxifen in the mouse brain: implications for fate-mapping studies using the tamoxifen-inducible Cre-loxP system. Front Cell Neurosci 10:243. 10.3389/fncel.2016.00243 27812322 PMC5071318

[B26] Vivacqua A, Bonofiglio D, Albanito L, Madeo A, Rago V, Carpino A, Musti AM, Picard D, Andò S, Maggiolini M (2006a) 17beta-estradiol, genistein, and 4-hydroxytamoxifen induce the proliferation of thyroid cancer cells through the g protein-coupled receptor GPR30. Mol Pharmacol 70:1414–1423. 10.1124/mol.106.026344 16835357

[B27] Vivacqua A, Bonofiglio D, Recchia AG, Musti AM, Picard D, Andò S, Maggiolini M (2006b) The G protein-coupled receptor GPR30 mediates the proliferative effects induced by 17beta-estradiol and hydroxytamoxifen in endometrial cancer cells. Mol Endocrinol 20:631–646. 10.1210/me.2005-0280 16239258

[B28] Yoshinobu K, Araki M, Morita A, Araki M, Kokuba S, Nakagata N, Araki K (2021) Tamoxifen feeding method is suitable for efficient conditional knockout. Exp Anim 70:91–100. 10.1538/expanim.19-0138 33055491 PMC7887626

[B29] Zhang Z, Park JW, Ahn IS, Diamante G, Sivakumar N, Arneson D, Yang X, van Veen JE, Correa SM (2021) Estrogen receptor alpha in the brain mediates tamoxifen-induced changes in physiology in mice. Elife 10:e63333. 10.7554/eLife.63333 33647234 PMC7924955

